# Formulation of catechol-containing adhesives for enhanced underwater bonding and workability

**DOI:** 10.1080/14686996.2025.2467617

**Published:** 2025-03-06

**Authors:** Cindy L. Atencio-Martinez, Alexandre Lancelot, Jonathan J. Wilker

**Affiliations:** a James Tarpo Jr. and Margaret Tarpo Department of Chemistry, Purdue University, West Lafayette, IN, USA; b School of Materials Engineering, Purdue University, West Lafayette, IN, USA

**Keywords:** Adhesive, biomimetic, formulation, mussels, polymer, underwater bonding

## Abstract

Catechol-containing polymers inspired by marine mussels have gained significant interest in recent years, leading to applications in several fields. Among these polymer systems, poly(vinylcatechol-styrene) (PVCS) has become a popular option due to its exceptional underwater adhesion strength, with readily available monomers and diverse synthetic routes being available. However, the translation of any novel adhesive chemistry from academic research to real-world applications can be challenging. Acrylates, epoxies, and urethanes were introduced to markets over half a century ago and remain dominant. However, bonding in wet environments remains lacking. The work presented here addresses this gap by focusing on the formulation of PVCS-based adhesives for conditions outside of the research lab. An emphasis was placed on handling properties when working underwater. A collection of different substrates were bonded together and several commercial glues provided benchmarks. Environmental conditions were studied to broaden the potential applications of PVCS adhesives in underwater settings. By optimizing formulations, we present an adhesive system that retains the superior underwater bonding of PVCS while also offering enhanced workability. This approach may help open the door to utilization of a new adhesive chemistry for underwater applications.

## IMPACT STATEMENT

This work advances biomimetic polymer formulations inspired by marine mussels, addressing current limitations in underwater adhesion and paving the way for practical, widely available underwater adhesives in various applications.

## GRAPHICAL ABSTRACT

**Figure:**
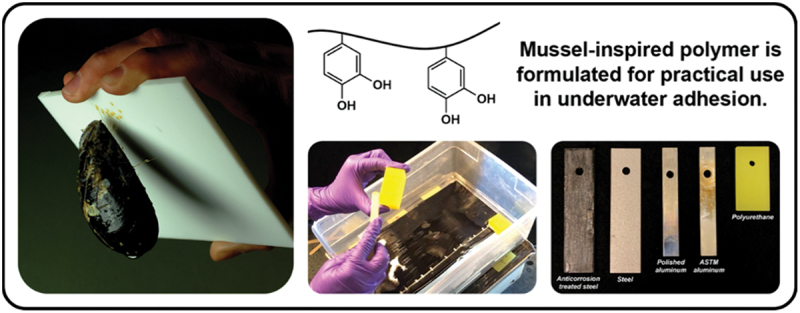

## 1. Introduction

Pendant catechol groups (i.e. dihydroxyphenyl) are found in the post-translationally modified amino acid 3,4-dihydroxyphenylalanine (DOPA). This catechol-containing amino acid provides marine mussels with their key ability to adhere onto surfaces underwater [1]. The binding mechanisms of proteins and synthetic polymers with catechols are well studied, although perhaps still not well understood. Multiple interactions with substrates have been observed, including noncovalent interactions and chemical bonding [2]. Catechol attachment to surfaces has been attributed to hydrogen bonding [3], π–π interactions [4], metal coordination [5], and covalent bonding [6]. Additionally, cohesive interactions are common, with cross-linking occurring through the catechol–catechol interactions [7]. Since JH Waite’s pioneering discovery of DOPA in mussel proteins during the 1980s [8,9], catechol-containing polymers have attracted significant research interest due to diverse chemistry and functionalities. Fundamental studies have explored potential applications in adhesives, coatings, drug delivery systems, and hydrogels for tissue engineering, amongst several other topics [10–13].

Robust adhesive hydrogels based on catechol chemistry have been developed to enhance the interaction of these soft materials with wet surfaces [14]. Collagen-catechol [15] and catechol-modified chitosan [16,17] hydrogels were synthesized for potential biomedical applications. Xu et al. [18,19] investigated the structural behavior of catecholamine methacrylamide gels exhibiting dual cross-linking via oxidation and coordination chemistry. A catechol-containing hydrogel rapidly fabricated under mild temperatures, reported by Jia and coworkers [20], showed excellent mechanical and electrical properties. Besides hydrogel formulations, the incorporation of catechols onto polymer coatings has also been explored. Han et al. [21] used catechol functionalities to immobilize polycations, achieving enhanced antimicrobial properties. Coatings with improved anticorrosion capabilities due to the hydrophobic properties and wet adhesion of catechol moieties have been described [22]. Additional catechol-containing coatings have been explored for applications in nanofiltration membranes [23].

Nevertheless, the primary application of catechol-containing polymers remains the development of innovative underwater adhesives, which have been synthesized through various routes and for a wide range of applications [24]. These adhesives have incorporated catechol moieties into different polymeric backbones to achieve strong bonding in both dry and underwater environments. For instance, poly(thioctic acylamino catechol) was recently synthesized by Shi et al. [25] and reported to display excellent strength aided by the hydrophobic nature of the polymer backbone, as well as via the formation of iron-catechol complexes responsible for cross-linking. Stronger adhesion of epoxy resins to aluminum and stainless steel substrates was achieved by preparing the surface of metal substrates with a poly[glycidyl methacrylate-co-(*N*-(3,4-dihydroxyphenethyl)methacrylamide] copolymer containing 15% catechol units [26]. A study by Kaur et al. [27] revealed the importance of catechol-binding groups in an elastomeric adhesive, resulting in enhanced dry and wet adhesion. Polyhydroxyurethane thermoset adhesives were shown to have improved mechanical and adhesive properties in dry conditions by incorporating 3.9 mol% dopamine to the formulation [28].

**Figure 1.**
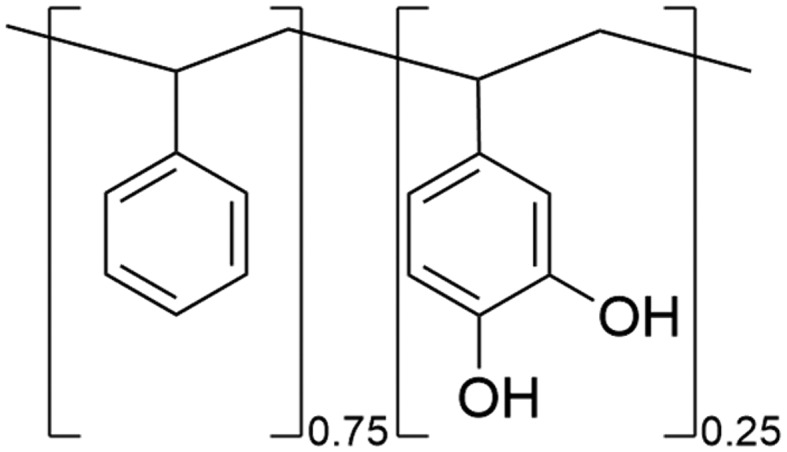
Chemical structure of poly(vinylcatechol-styrene).

We appear to be approaching the point at which practical use of this new class of adhesive chemistry is viable. However, there are not yet any catechol-based adhesives on the market. Reaching such a point still requires some work. In particular, the selection of a well-defined catechol-containing polymer system, facile synthesis, tailored handling properties, and evaluation of performance closer to real-world uses, beyond the confines of the research lab, are still needed. Defining a suitable polymer system requires consideration of several factors, including monomer compatibility, availability at large scales, reproducible synthetic routes, low cost, and validation by a broader scientific community. This extended validation often needs the development and optimization of the chosen polymer system over a substantial period of time. In this context, poly(vinylcatechol-styrene) (PVCS) (Figure 1) is emerging as a promising candidate. Catechol and styrene monomers exhibit high compatibility due to the structural similarity between the catechol moiety and the phenyl ring in styrene [29]. This close resemblance facilitates monomer copolymerization, minimizing potential disruptions to the final polymer architecture [30]. Furthermore, the established commercial availability and cost-effectiveness of styrene provides an attractive chain host for catechol incorporation.

Our research group has studied poly(vinylcatechol-styrene) for over 15 years [30]. While our laboratory has made significant contributions to its development [29–37], many other research teams have also explored several aspects of PVCS. Numerous studies have investigated the anionic polymerization of PVCS with varying monomer compositions [30,33]. Catechol-containing monomers protected with acetal groups have been homo- and copolymerized with styrene by means of anionic polymerization [38,39]. Saito and coworkers [40,41] reported the reversible addition – fragmentation chain transfer (RAFT) polymerization of poly(vinylcatechol-*block*-styrene). RAFT has also been used for the homopolymerization of dimethoxystyrene [42], as well as biobased protected vinyl catechol monomers, specifically caffeic acid [43] and ferulic acid [44]. Pino et al. [45] synthesized a catechol-containing styrenic block copolymer via nitroxide-mediated controlled radical polymerization, and Yabu and Nagano [46] reported the copolymerization of block and random poly(vinylcatechol-styrene) through RAFT and free radical polymerization, respectively. Beyond synthetic chemistry, computational modeling studies by Dunbar and Keten [47] strengthen the case for PVCS to be a versatile adhesive platform. Their work simulated the short time dynamic properties of PVCS across various temperatures through an energy normalization approach.

While these studies demonstrate diverse synthetic routes for catechol-styrene copolymers, many are limited in scale. Nonetheless, we have recently reported the radical-initiated suspension polymerization of PVCS, allowing the synthesis of polymer batches of up to 60 g [35]. This suspension copolymerization method offers advantages due to inherent safety, ease of use, and cost-effectiveness compared to traditional radical and anionic approaches. Moreover, synthesis of PVCS with higher molecular weights, achieving number average molecular weights (M_n_) of ~40 kDa and weight average molecular weights (M_w_) of ~160 kDa were accessible. Prior studies have shown that, under specific conditions, a PVCS-based adhesive can exhibit superior underwater bonding across different types of substrates compared to a group of 10 commercially available adhesives [33]. These results suggest the potential of PVCS to be a specialized underwater adhesive with competitive performance within relevant markets.

This current study presents a systematic investigation of factors influencing the formulation of poly(vinylcatechol-styrene) adhesives for optimal underwater adhesive bonding. Emphasis was placed on handling, reproducibility, and scalability, examining various parameters such as solvent selection, polymer concentration, filler type and incorporation, water temperature, salinity, pH, and physical movement. Adhesion performance was evaluated using epoxy-coated steel bonded to polyurethane substrates via lap shear testing. The successful adhesion between these materials addresses a critical need in diverse industrial applications requiring underwater bonding of strong, flexible, and corrosion-resistant materials. Examples of such applications include high-pressure composite pipes [48], shock and vibration absorbing infrastructure [49–52], and storage tanks [53,54]. Polyurethane offers flexibility [55], corrosion resistance [56], and insulation [57]. Steel provides strength, rigidity, and excellent overall mechanical properties, although underwater conditions require coating of steel to prevent corrosion. Bonding these materials together leverages the unique advantages of each substrate. Additional substrate pairs were also bonded together including coated steel-to-coated steel, polyurethane-to-polyurethane, steel-to-steel, and aluminum-to-aluminum. Several commercial glues were studied to provide suitable performance benchmarks. The peel adhesion properties of PVCS on flexible adherends, such as etched Teflon® and polypropylene, were also evaluated. The results presented in this report achieve robust bonding and will further contribute to the growing evidence supporting the potential of poly(vinylcatechol-styrene), while also advancing the field of adhesive chemistry beyond the limited catalog of acrylates, epoxies, and urethanes that has dominated the market for over five decades. We may be soon at a place where biomimetic adhesives can enter the marketplace.

## 2. Materials and methods

### 2.1. Materials

Solvents were purchased from Sigma-Aldrich or Fisher Scientific and used without additional purification. Calcium carbonate (Vicron ® 15–15) with average particle size of 3.5 µm was donated by Specialty Minerals, Inc. (New York, NY). Recycled tire rubber, acrylate-butadiene rubber (ABR) mesh size 80–99, was obtained from Entech, Inc. (Middlebury, IN).

### 2.2. Polymer synthesis

Poly(vinylcatechol-styrene) was synthesized following a previously reported method [35]. Briefly, a radical-initiated suspension copolymerization in water was employed to copolymerize 3,4-dimethoxystyrene and styrene using poly(vinyl alcohol) and sodium dodecyl sulfate as suspension agents, and benzoyl peroxide as thermal initiator. The resulting poly(3,4-dimethoxystyrene-styrene) was demethylated using iodocyclohexane to yield poly(vinylcatechol-styrene) as a light brown powder. ^1^H NMR (CDCl_3_, 300 MHz): δ (ppm) 7.07 (m, 2.28 h, 3 h_Ar-styr_), 6.58 (m, 1.76 h, 2 h_Ar-styr_+1 h_Ar-cat_), 6.00 (m, 0.48 h, 2 h_Ar-cat_), 1.78 (m, 1 h, −C*H*Ph-CH_2_−), 1.44 (m, 2 h, −CHPh-C*H*_2_−). A representative ^1^H NMR spectrum of poly(vinylcatechol-styrene) is shown in the Supplementary Information, Figure S1. Gel Permeation Chromatography (GPC) of poly(3,4-dimethoxystyrene-styrene) was conducted using an Agilent 1260 Infinity II liquid chromatography system (Agilent Technologies, Santa Clara, CA), equipped with two WAT044228 Styragel Waters columns (Waters Corporation, Milford, MA) in series. Tetrahydrofuran was used as the mobile phase. The analysis yielded a number average molecular weight (M_n_) of 39.4 kDa, and a weight average molecular weight (M_w_) of 156.2 kDa. A representative GPC chromatogram is shown in the Supplementary Information (Figure S2).

### 2.3. Substrates preparation

Steel panels HR SP5 (12” × 6” × 1/8”, 4” × 1” × 1/8”, and 6” × 2” × 1/8”) were obtained from KTA-Tator, Inc (Pittsburgh, PA) and coated with a 2-part epoxy coating, Hycote 151, purchased from Somay Manufacturing, Inc. (Miami, FL). Steel substrates were coated to prevent corrosion in aqueous environments. Epoxy coatings effectively address this challenge, offering exceptional adhesion to steel, superior chemical resistance, and ease of application [58]. Polyurethane sheets (1” × 24” × 24” and 1/4”× 24” × 24”) were purchased from United States Plastic Corp. (Lima, OH) and cut into 2” × 1” × 1” and 2” × 1” × 1/4” pieces using a 10” Saw Stop Table Saw (SawStop LLC, Tualatin, OR) with a Diablo 10” 90 teeth saw blade. Three feet long 6061 aluminum sheets (1/8”× 1/2”) were obtained from McMaster-Carr (Aurora, OH) and cut into 3.5” long pieces using a Wysong Metal Shear (Wysong Equipment, Pewaukee, WI). Aluminum substrates were polished using a buffing wheel with a Tripoli brown buffing compound followed by a green buffing compound. Subsequently, the substrates underwent solvent cleaning with hexanes, acetone, methanol, and water. Etching of aluminum substrates was performed following the ASTM D2651 method [59], utilizing boiling base and acid baths, followed by cleaning steps in methanol and boiling water. Etched Teflon® (48” × 24” × 0.02”) and polypropylene (45” × 24” × 0.02”) sheets were obtained from McMaster-Carr (Aurora, OH) and cut into 7” × 1” strips using a paper trimmer.

### 2.4. Water preparation

Adhesion experiments were set up in artificial seawater prepared at least 24 h prior with a specific gravity of 1.025, using Marine Environment® dual-phase formula.

### 2.5. Lap shear adhesion testing

Lap shear bonding was employed for all adhesion tests in this study. Each experiment utilized seven replicate samples. For coated steel bonded to polyurethane, seven 1” × 1/2” rectangles were marked on 12” × 6” × 1/8” coated plates. The coated steel surfaces were lightly sanded with 80-grit aluminum oxide abrasive paper and cleaned with butyl alcohol before submersion in artificial seawater. The designated adhesive, either a formulated solution or commercial glue, was applied to the submerged steel in the marked 0.5” × 1” area. Individual 2” × 1” × 1” polyurethane pieces, also submerged, were then bonded to the marked areas to achieve bonding underwater. Coated steel-to-coated steel (4” × 1” × 1/8”) and polyurethane-to-polyurethane (2” × 1” × 1/4”) bonding followed the same procedure with the same overlap area. Aluminum substrates (3.5” × 1/2” × 1/8”) were prepared according to a modified version of the ASTM D1002 standard for lap shear bonding [60]. A centered hole with a diameter of 1/4” was drilled 7/8” from one end of each substrate, and a bonding area of 1/2” × 1/2” was established for the adhesive overlap. In most cases, a 20 g weight was applied on top of the adhesive joints to aid curing. Unless stated otherwise, all samples were cured underwater for 3 days. Following underwater curing, samples were removed from the artificial seawater and tested to failure using an Instron 5544 materials testing system (Instron Corporation, Norwood, MA) equipped with a 2 kN load cell. Each sample was subjected to a constant crosshead speed of 2 mm/min. The maximum force required to break the bond was recorded. Lap shear strength was determined by dividing the maximum force by the bonded overlap area for each sample. The data were subsequently averaged and error bars represent 90% confidence intervals.

### 2.6. Viscosity experiments

Rheological properties of poly(vinylcatechol-styrene) solutions at various concentrations were investigated using a TA Instruments Discovery HR30 Hybrid Rheometer (TA Instruments, New Castle, DE). Control experiments were performed on polystyrene samples (Sigma-Aldrich, average M_w_ = 90,000 g/mol) at corresponding concentrations. Viscosity measurements were obtained using a Peltier recessed concentric cylinder geometry at a constant temperature of 25°C. A solvent trap was employed throughout the experiments to minimize solvent evaporation. Triplicate measurements were conducted at each concentration with a shear rate sweep from 0.01 to 100 s^−1^, recording five data points per decade.

### 2.7. Imaging

Failed bond surfaces were characterized using optical microscopy and scanning electron microscopy. Optical images were acquired using an Olympus BX-51 Optical Microscope (Olympus Corporation, Tokyo, Japan). High-resolution micrographs of the fracture surfaces were obtained using a Quanta 3D FEG dual-beam electron microscope (FEI Company, Hillsboro, OR).

### 2.8. Water temperature range study

For room temperature experiments, joints were set up in the laboratory without further temperature modification. For experiments at 3°C, artificial seawater was cooled down in a refrigerator set to 3°C and verified with a thermometer. After reaching the desired temperature, seawater was removed from the refrigerator, experiments were set up immediately, and samples were returned to the refrigerator until testing. For experiments at 60°C, seawater was heated using an Anova Culinary Sous Vide Precision Cooker Nano 3.0 and maintained at that temperature until testing.

### 2.9. Contact angle goniometry

Contact angle measurements were conducted using an Attension Theta optical tensiometer (Biolin Scientific, Espoo, Finland). For each measurement, a 3 µL droplet of deionized water was deposited onto the substrate using a micropipette, and the tensiometer captured images of the droplet at video rates. The images were subsequently analyzed with the OneAttension software in sessile droplet mode, employing the Young-Laplace equation. Each contact angle measurement is based on the average of 152 data points, with four measurements taken on each sample. Reported errors correspond to one standard deviation.

### 2.10. 90° peel testing

Coated steel substrates (6” × 2” × 1/8”) were lightly sanded with 80-grit aluminum oxide abrasive paper, cleaned with butyl alcohol, and submerged under artificial seawater. Adhesive formulations were then applied to a 5” × 1” area of etched Teflon® or polypropylene strips (7” × 1” × 0.02”) in air and bonded to the submerged coated steel substrates. A 2” section of the flexible adherend was left unbonded. A 380 g weight was placed on top of the strips, while the adhesive cured underwater for 7 days. Four samples were prepared for each adhesive formulation and flexible substrate type. After curing, the specimens were removed from the water and placed into the 90° peel test fixture of an Instron 3340TM–30 testing machine (Instron Corporation, Norwood, MA) equipped with a 2 kN load cell. The unbonded 2” end of the flexible adherend was bent perpendicular to the steel substrate and clamped into the grip. The samples were then peeled at a constant crosshead speed of 254 mm/min, following the ASTM 6862 standard [61].

## 3. Results and discussion

### 3.1. Solvent study

Previous underwater work with PVCS adhesives used chloroform for the solvent of choice given the ability to prevent polymer precipitation during underwater application due to immiscibility with water [33]. The high density of chloroform also provided the practical benefit of having formulations remain at the bottom of tanks. However, toxicity associated with chloroform poses an obstacle to commercialization. We explored alternative solvents that maintain the desired adhesive properties of PVCS while prioritizing industrial viability. To facilitate initial investigations and ensure consistency with established protocols for underwater adhesive applications, a PVCS concentration of 0.3 g/mL was selected in a given solvent, consistent with reports by North et al. [33] and Lancelot et al. [35].

Acetone was initially explored due to good solubility for PVCS. However, the complete miscibility of acetone with water [62] led to rapid solvent dissipation underwater. Since PVCS is not water-soluble, polymer precipitation was observed immediately upon acetone dissipation. The interaction between PVCS and the substrate surface was hindered, preventing formation of adhesive bonds. Methyl ethyl ketone (MEK) was explored for a potential replacement due to similar properties with acetone and good solvating abilities for PVCS. Unlike the complete miscibility of acetone with water, MEK exhibits only partial water compatibility [63]. We found that introduction of hexanes reduced the overall miscibility with water, thereby hindering water permeation into the adhesive joint during underwater application and curing. This strategy did not impact the performance of the PVCS adhesive too much (Supplementary Information, Figure S3). An investigation was conducted to determine the optimal hexanes concentration, revealing that a 9:1 v/v MEK/hexanes ratio yielded the most favorable results (Supplementary Information, Figure S4).

To identify the chemical components remaining on substrates after lap shear testing, and quantify the water content within the adhesives, samples were analyzed using ^1^H NMR spectroscopy after failure. Two distinct sample preparation methods were employed. First, an immediate test was designed so that samples were retrieved directly from the underwater environment and subjected to lap shear testing without any drying step. This approach captured the entire adhesive layer, potentially including interfacial water, for subsequent analysis. A second test was carried out by retrieving samples from the underwater environment and allowing them to dry for 1 h under ambient conditions before lap shear testing. This dried adhesive polymer was then isolated for ^1^H NMR analysis. Data obtained from the ^1^H NMR analyses are summarized in Table 1. The initial molar composition of PVCS and the corresponding solvents used in the formulations are presented, as well as the molar ratios of PVCS, solvent, and water measured after both the immediate test and the 1-h dried test for each sample. Figures S5-S10 in the Supplementary Information show representative ^1^H NMR spectra for these samples.

**Table 1.** ^1^H NMR analysis of water content in adhesive samples using different solvents.

		PVCS in Chloroform			PVCS in MEK			PVCS in MEK/hexanes (9:1)			
		PVCS	Chloroform	Water	PVCS	MEK	Water	PVCS	MEK	Hexanes	Water
Initial composition	Normalized moles	1.00	8.28	–	1.00	1.68	–	1.00	1.52	0.11	–
	Percentage (%)	11	89	–	37	63	–	38	58	4	–
Immediate test	Normalized moles	1.00	–	0.70	1.00	0.22	13.03	1.00	0.14	0.03	1.92
	Percentage (%)	59	–	41	7	2	91	32	5	1	62
One-hour dried test	Normalized moles	1.00	–	0.56	1.00	0.23	0.56	1.00	0.20	0.10	0.84
	Percentage (%)	64	–	36	56	13	31	47	9	5	39

Analysis of samples retrieved immediately after testing revealed an influence of solvent selection on water uptake into the adhesive. Samples prepared with the MEK/hexanes mixture exhibited a 29% reduction in water content compared to those dissolved into solely MEK. Furthermore, samples prepared with chloroform displayed the lowest water content. This observation can be attributed to the inherent hydrophobicity of chloroform and hexanes, which likely minimized water permeation into the adhesive. Samples allowed to dry for 1 h before testing showed similar water content across all solvent formulations. This result suggests that the permeation of water into the adhesive bulk was similar regardless of the initial solvent composition. Based on the combined analysis of these results and the adhesion performance of the formulations, a MEK/hexanes (9:1) solvent system was selected for further investigation in subsequent studies.

Additional qualitative experiments were conducted to investigate the effect of PVCS binding to coated steel substrates. Water contact angles were measured on both the coated steel substrates and the residual adhesive polymer layer remaining after lap shear testing. To further examine the impact of chemical binding to the surface, the remaining adhesive layer was scraped from the substrate, and contact angles were measured again on the coated steel substrate. Figure S11 in the Supplementary Information shows representative images of the water contact angles for each of the three surfaces. The water contact angle on coated steel was found to be 74 ± 5°, compared to 93 ± 4° when the water droplet was placed on the PVCS layer. After scraping the polymer from the coated steel surface, the water contact angle was measured at 92 ± 4°. The higher contact angles measured on the PVCS adhesive layer, compared to the coated steel, can be attributed to greater hydrophobicity of poly(vinylcatechol-styrene) relative to epoxy coatings. The hydrophobicity of PVCS arises from the incorporation of 25 mol% catechol into the 75 mol% hydrophobic styrene backbone. Similar contact angles observed on the PVCS layer and the scraped surface suggest that PVCS remains chemically bonded to the coated steel.

### 3.2. Polymer concentration

Polymer concentration in solution is a critical parameter in adhesive development, influencing viscosity, wetting behavior, ease of application, and, ultimately, overall performance [64]. These factors become particularly important for an adhesive that specializes in underwater bonding. Although prior studies have demonstrated the competitiveness of PVCS-based adhesives, practical considerations like ease of application in wet environments must also be addressed. Here, we observed that the previously used 0.3 g/mL solution consistency was thin and rolled away, potentially hindering application in quotidian scenarios. To address this challenge, we investigated the underwater adhesion performance of PVCS solutions at various concentrations, ranging from 0.3 g/mL to the solubility limit of 1.2 g/mL, when PVCS was dissolved into chloroform, MEK, or MEK/hexanes (9:1). This study strived to optimize underwater adhesion strength while minimizing the required polymer content and, most importantly, ensuring user-friendly application.

**Figure 2.**
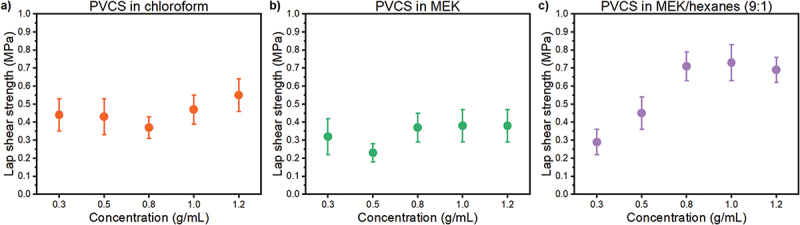
Effect of polymer concentration in underwater adhesion when dissolved into a) chloroform, b) methyl ethyl ketone, and c) a mixture of methyl ethyl ketone and hexanes (9:1).

Figure 2 shows lap shear adhesion strength as a function of polymer concentration in solution for the three different solvents. Bonding of PVCS dissolved into chloroform (Figure 2a) and MEK (Figure 2b) exhibited similar trends, with consistent adhesion observed throughout the range of concentrations. PVCS dissolved into the MEK/hexanes (9:1) mixture exhibited the highest adhesion values, with increased bonding at higher concentrations (Figure 2c). Solutions at 0.8 g/mL and above displayed favorable consistency for underwater application, allowing for easy manipulation and adhesion to substrates with diverse shapes and geometries. These formulations also demonstrated minimal dripping in a vertical setting, a crucial characteristic for many applications. The highest lap shear strength of 0.73 ± 0.10 MPa was achieved with a 1.0 g/mL concentration in MEK/hexanes, although a 95% confidence ANOVA revealed no statistically significant difference in adhesion performance between 0.8, 1.0, and 1.2 g/mL concentrations, respectively, 0.71 ± 0.08 MPa, 0.73 ± 0.10 MPa, and 0.69 ± 0.07 MPa. In order to prioritize both adhesion strength and material efficiency, a concentration of 0.8 g/mL was selected for further studies.

**Figure 3.**
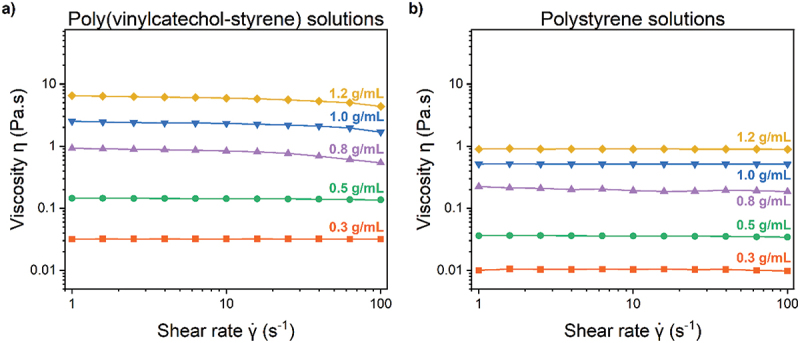
Viscosity versus shear rate of a) poly(vinylcatechol-styrene) solutions and b) polystyrene solutions at varying concentrations.

To elucidate the handling properties, viscosity experiments were performed on PVCS solutions at varying concentrations prepared in 9:1 MEK/hexanes mixtures. Flow curves for the solutions are presented in Figure 3a. A clear progressive increase in viscosity with increasing concentration was observed. Solutions at 0.3 and 0.5 g/mL displayed shear-independent viscosity behavior across the range of shear rates investigated. Solutions at concentrations ranging from 0.8 g/mL to 1.2 g/mL exhibited moderate shear thinning tendencies, evident by decreases in viscosity at higher shear rates, characteristic of non-Newtonian fluids. The shear thinning behavior was better visualized in the linear plots depicted in the Supplementary Information (Figure S12). For comparison purposes, control experiments were conducted using commercially available polystyrene (PS) – i.e. no catechol – dissolved in the same solvent mixture at equivalent concentrations. The corresponding flow curves are shown in Figure 3b. The PS solutions displayed a similar trend of increasing viscosity with concentration compared to PVCS solutions. However, PS solutions consistently exhibited lower viscosities than PVCS solutions at corresponding concentrations. We attribute this observation to two primary factors. First, the polymers possess differing molecular weights. The average molecular weight of PVCS was approximately 160 kDa, whereas the commercially obtained PS had an average molecular weight of 90 kDa. Previous literature reports demonstrated that increasing the molecular weight of polystyrene resulted in higher viscosity values at the investigated shear rates [65]. Moreover, intra- and intermolecular interactions, such as hydrogen bonding between catechols’ hydroxyl groups, are known to promote chain entanglements, significantly impacting viscosity [66]. Consequently, PS solutions may exhibit lower viscosity values at identical shear rates compared to PVCS at equivalent molecular weights. Additionally, at a given shear rate (e.g. 10 s^−1^), the viscosity of polystyrene solutions increased linearly with concentration. In contrast, poly(vinyl catechol-styrene) solutions exhibited a non-linear behavior, consistent with the characteristics of adhesive polymers, which can interact with the recessed concentric cylinder geometry of the rheometer and undergo hydrogen bonding (Supplementary Information, Figure S13) [67]. Considering these factors, the behavior of PVCS solutions relative to PS solutions fell within an expected range. These findings show a positive correlation between solution concentration and viscosity. The observed shear-thinning behavior at elevated shear stresses suggests potential improvements in adhesive handling while also reducing dripping and sagging when not being manipulated (i.e. at lower shear rates), compared to lower concentration formulations.

### 3.3. Fillers

Fillers are incorporated into adhesives to modify both mechanical and physical properties. These modifications may – or may not – include improved overall strength and durability, enhanced crack dissipation, distribution of mechanical stresses, increased heat resistance, and better ease of application [68]. Note, however, that fillers may also inhibit these desirable properties. Additionally, fillers offer an economic benefit by reducing the overall cost of formulations [69]. Inorganic fillers, such as carbonate particles, and polymeric fillers like rubber powders are commonly used [70]. Previous studies have demonstrated the advantages of using fillers in adhesives. For instance, we increased the dry adhesion of a catechol-containing polymer from 4 to 5 MPa on aluminum substrates by adding calcium carbonate [71]. Addition of CaCO_3_ with a particle size of 3.5 µm resulted in greater adhesion improvement compared to calcium carbonate with a particle size of 70 nm, a phenomenon also observed in previous studies using poly(vinylcatechol-styrene) formulations in dry conditions [32]. Nanometer-scale particles require surface modification, often via coating with stearic acid, to prevent agglomeration and maintain particle size. However, this modification may hinder polymer–filler interactions and reduce the overall adhesion [72]. Rubber particles, in particular, have been employed to toughen polymer matrices due to dissipation of mechanical stresses [73]. Ong et al. [74] achieved ca. 180% increase in adhesion of plywood by adding 13 wt.% waste rubber powder into a melamine urea formaldehyde glue. For the underwater applications here, the hydrophobic nature of rubber particles was particularly appealing. Inclusion of rubber may discourage water from penetrating into the material.

**Figure 4.**
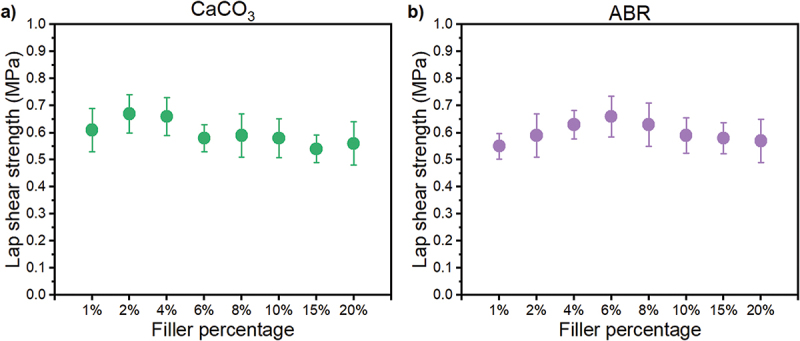
Underwater adhesion performance of PVCS-based glues upon addition of a) calcium carbonate, and b) recycled ABR rubber.

Here, we investigated the effect of incorporating 3.5 µm CaCO_3_ and recycled tire rubber (acrylate-butadiene rubber, ABR) at loadings of 1–20 wt.% into PVCS formulations. Both fillers were uniformly dispersed into the polymer matrices prior to application, evidenced by visual inspection and optical microscopy, as seen in the Supplementary Information, Figure S14. The addition of 2 wt.% CaCO_3_ resulted in a mild maximum underwater lap shear strength of 0.67 ± 0.07 MPa (Figure 4a). Statistical analysis of variance revealed no significant change in adhesion for the unfilled polymer at the 95% confidence level. However, it may be relevant to consider that the hydrophilic character of calcium carbonate could promote water migration into the adhesive over extended periods. This potential water ingress might weaken bonds over time. Consequently, the addition of ABR was also examined. The presence of 6 wt.% ABR to PVCS yielded a maximum lap shear strength of 0.66 ± 0.08 MPa (Figure 4b). Here, too, the filler changes to adhesion were not statistically significant. Perhaps, ABR can enhance the overall hydrophobicity of the polymer system.

Scanning electron microscopy (SEM) and optical microscopy images were taken on substrates after lap shear testing to failure to investigate the influence of fillers on the mechanical behavior of PVCS. Coated steel-to-polyurethane specimens exhibited adhesive failure, with all of the polymeric material remaining on the steel substrate (Supplementary Information, Figure S15a). Control experiments were conducted with the optimized PVCS formulation, alongside those formulated with the fillers that achieved the highest adhesion values, i.e. PVCS +2 wt.% CaCO_3_ and PVCS +6 wt.% ABR.

**Figure 5.**
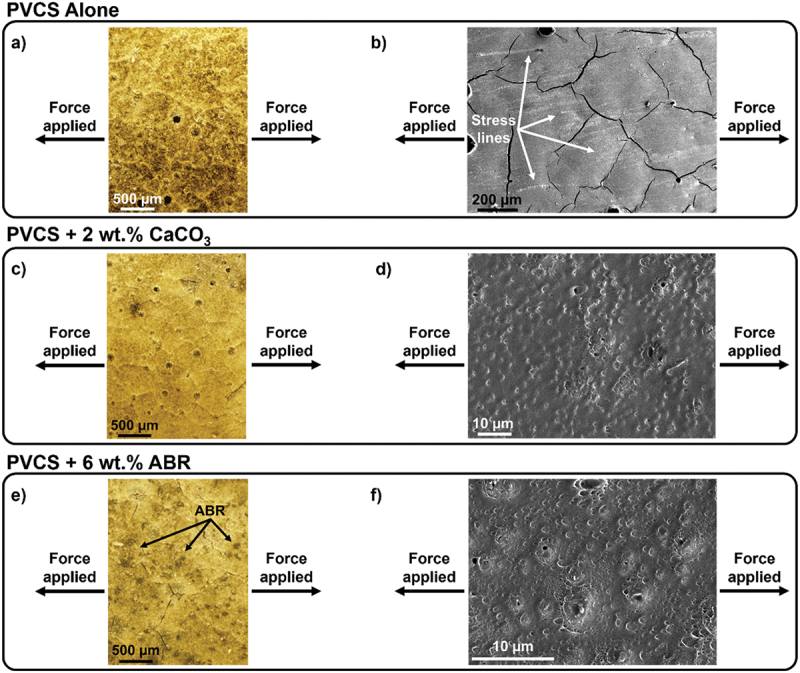
Optical microscopy and SEM images of adhesive failure on coated steel surfaces of a) & b) PVCS alone, c) & d) PVCS +2 wt.% CaCO_3_, and e) & f) PVCS +6 wt.% ABR.

Brittle fracture characteristics were evident in the coated steel-to-polyurethane adhesive joints for unfilled PVCS (Figure 5). Force-extension plots displayed sharp curves, also indicative of brittle behavior, particularly in the PVCS alone case (Supplementary Information, Figure S16). Additionally, optical micrographs (Figures 5a,c,e) revealed cracks within the adhesive surface. The SEM micrographs of PVCS alone (Figure 5b) also identified the presence of stress lines, corroborating the brittle fracture mechanism. When filled with either CaCO_3_ or ABR, fracture lines were not observed. Furthermore, the force–extension curves showed less sudden failure. Thus, the fillers appear to have toughened the material to some degree. The SEM analysis did not show CaCO_3_ or ABR particles within the superficial adhesive layer (Figure 5d,f). However, ABR particles were detectable by optical microscopy. These particles were likely coated by the polymer matrix, suggesting minimal influence on the adhesive-substrate failure at the interface. Due to small sizes falling below the resolution limit, CaCO_3_ particles were not observed via optical microscopy. The absence of CaCO_3_ observed in SEM micrographs further indicated a lack of major presence at the surface, similar to the ABR particles.

**Figure 6.**
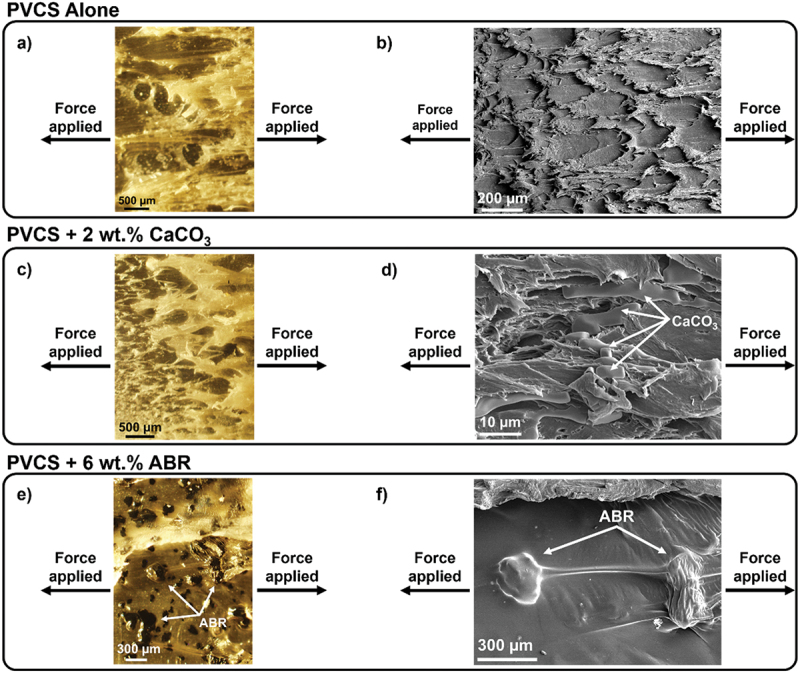
Optical microscopy and SEM images of cohesive failure on polished aluminum surfaces of a) & b) PVCS alone, c) & d) PVCS +2 wt.% CaCO_3_, and e) & f) PVCS +6 wt. % ABR.

To contrast the behavior of the fillers under regimes dominated by strong adhesive versus cohesive forces, cohesive failure was induced by repeating the experiments using polished aluminum substrates. Bonded polished aluminum adherends exhibited ductile cohesive failure (Supplementary Information, Figure S15b). This behavior was reflected in the force-versus-extension curves, which displayed a less sudden break as well as higher areas-under-the-curve (i.e. work of adhesion) and extension values compared to the coated steel-to-polyurethane joints (Supplementary Information, Figures S16 versus S17). A textured structure of the polymer matrix was seen, with stretching in the direction of pulling (Figure 6). These micrographs further supported the cohesive and ductile nature of the failure mode on aluminum. The SEM images revealed CaCO_3_ particles dispersed within the various layers of the polymer matrix (Figure 6d). For a control, SEM was performed on isolated particles, without any polymer, to confirm the identity to be CaCO_3_ (Supplementary Information, Figure S18). The ABR particles were predominantly located on the adhesive surface while also exhibiting a thin layer of PVCS coated on top (Figure 6e,f). The disparity in particle behavior and distribution may be attributed to the size difference between fillers. The average particle size of 3.5 µm for CaCO_3_ may have enabled sufficient mobility during testing to become embedded in the different layers of the polymer matrix while force was exerted during testing. The larger ABR particles (150–175 µm) might have experienced hindered movement. Differences in particle surface energies may also have influenced this behavior.

### 3.4. Benchmarks and adhesion in different substrates

To establish a baseline for the underwater adhesion performance of the preferred PVCS formulation (i.e. PVCS dissolved into a mixture of MEK/hexanes (9:1) at a concentration of 0.8 g/mL), commercially available adhesives with diverse chemistries were evaluated for abilities to bond coated steel-and-polyurethane underwater. A selection encompassing established adhesive technologies was chosen, including a two-part epoxy specifically formulated for underwater use (Mr. Sticky’s), a solvent-free, rubberized waterproof adhesive (Flex Glue), a water-activated polyurethane adhesive (Gorilla Glue), and a cyanoacrylate adhesive (Super Glue). Adhesion tests were conducted on bonds between like substrates: coated steel-to-coated steel, polyurethane-to-polyurethane, and also uncoated steel-to-uncoated steel. Aluminum substrates were also examined given the common use of this material in different industrial settings. Etched aluminum and polished aluminum were further included in order to have a better understanding of different surface types and finishes.

Table 2 shows the underwater adhesion performance of PVCS across different substrates, compared to commercial glues. Among the five adhesives evaluated, the PVCS-based formulation displayed the strongest adhesion for coated steel-to-polyurethane bonds. Mr. Sticky’s surpassed all adhesives, including PVCS, when bonding steel and coated steel substrates. These results align with the well-known strength of epoxy within the spectrum of adhesive chemistries [75]. Bonding low surface energy substrates like polyurethane poses a challenge for many or all adhesives, evidenced here by the low comparable performance observed across the commercially available options and the PVCS formulation. In contrast, the PVCS-based adhesive exhibited exceptional strength on both polished and etched aluminum. This superior performance compared to commercially available adhesives likely stems from the presence of unique catechol moieties within the PVCS polymer. These functional groups are known to promote favorable interactions with metal surfaces [71]. Furthermore, the enhanced bonding strength observed for etched aluminum, at least double that of other adhesives, suggests a potential contribution from mechanical interlocking between the PVCS adhesive and roughened topography of the etched surface [29].

**Table 2.** Underwater adhesion strength of PVCS compared to commercial glues on different substrates. The highest strengths for a given substrate are in bold.

Substrate	PVCS (MPa)	Mr. Sticky’s (MPa)	Flex Glue (MPa)	Gorilla Glue (MPa)	Super Glue (MPa)
Coated steel – Polyurethane	**0.7 ± 0.1**	0.5 ± 0.1	0.3 ± 0.1	0.4 ± 0.1	~0
Coated steel	1.2 ± 0.1	**4.8 ± 1.5**	0.8 ± 0.3	0.1 ± 0.1	0.1 ± 0.1
Polyurethane	0.1 ± 0.0	**0.3 ± 0.1**	0.2 ± 0.0	0.1 ± 0.0	0.1 ± 0.0
Steel	0.7 ± 0.2	**3.2 ± 0.4**	0.8 ± 0.1	0.1 ± 0.1	~0
Etched aluminum	**1.2 ± 0.4**	0.3 ± 0.1	0.5 ± 0.2	~0	~0
Polished aluminum	**2.3 ± 0.7**	1.4 ± 0.3	2.0 ± 0.2	1.4 ± 0.3	~0

### 3.5. Study of environmental conditions

Long-term robust underwater adhesion, when achieved, will offer advancements for scientific and technological applications in marine environments. Such a breakthrough could enable efficient subsea repair of critical infrastructure, facilitating longer lifespans, and reduced downtime for offshore platforms and pipelines [76]. Long-lasting underwater adhesives could also help deploy marine sensors for extended periods, enabling the acquisition of crucial oceanographic data for climate monitoring, marine species tracking, resource exploration, and ecosystem understanding [77–79]. However, these applications require the use of adhesive formulations that can adapt to diverse and constantly changing environments. When evaluating adhesives for real-world underwater applications, several factors must be considered, including water temperature, movement, long-term performance, and environmental conditions such as salinity and pH. These factors were investigated to assess the performance of poly(vinylcatechol-styrene) adhesives under varying conditions.

First, we investigated the adhesion performance of the optimized PVCS formulation under three distinct water temperatures: 3°C, room temperature (~25°C), and 60°C. Water at 3°C mimicked deep ocean environments, and 60°C water served as approximating conditions encountered in pipes within oil rigs and production facilities [80]. The lap shear strength was assessed after 1, 3, 7, and 28 days underwater. This selection reflected the potential for weakened adhesion over time due to degradation of the adhesive and increased water absorption at the joint [81]. Furthermore, this approach aligns with understanding how polymeric materials in service environments may be subjected to diverse environmental factors [82]. Elevated temperatures are a critical parameter for predicting polymer aging [83]. Concerns in such scenarios can include potential solvent loss over time as well as proximity of operating temperature to the glass transition temperature (Tg) of the polymer, which can impact mechanical performance. The reported Tg of PVCS is approximately 120°C [35].

**Figure 7.**
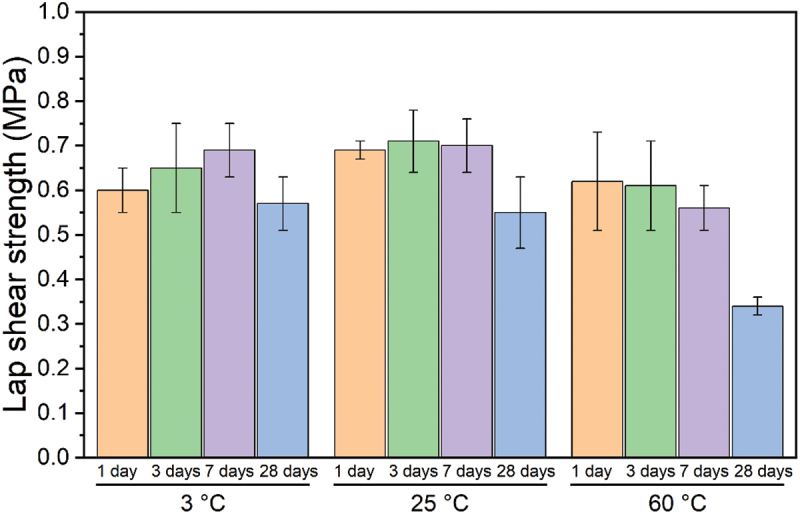
Effect of water temperature in adhesion of PVCS over time.

Figure 7 shows results from the temperature range study. At room temperature, adhesion remained constant after 1, 3 and 7 days of application, followed by a decrease from 0.70 ± 0.06 MPa to 0.55 ± 0.08 MPa after 28 days. This trend suggested good initial bond formation followed by potential slow degradation processes at longer time scales. Specimens tested at 3°C exhibited a gradual increase in adhesion strength over the first week. This behavior may be attributed to the combined effects of lower temperatures, reducing polymer chain mobility, and slow solvent loss rates. These phenomena potentially extended the curing time of the adhesive. After 28 days, the adhesion strength achieved at 3°C was comparable to the bonding observed at room temperature after the same period of time. Testing at an elevated temperature of 60°C revealed a potential decrease in adhesion values compared to room temperature and 3°C water, and an overall trend of decreasing strength over time. While the reported glass transition temperature (Tg) of PVCS is twice the evaluation temperature here, the hot water might have enhanced the mobility of polymer chains, potentially hindering their ability to form strong interfacial interactions. Furthermore, the adhesion strength of the PVCS formulation at 60°C decreased from 0.62 ± 0.11 MPa at 1 day to 0.34 ± 0.02 MPa after 1 month, potentially due to the initiation of oxidation or thermal decomposition of the polymer at prolonged high temperatures [84]. Nonetheless, the ability of PVCS to form quantifiable, durable bonds for 4 weeks under what can be considered harsh conditions (under artificial seawater, at 60°C) remains remarkable. These results illustrate the relevance of considering both temperature and immersion time for optimizing the underwater adhesion performance of adhesives for various applications.

**Figure 8.**
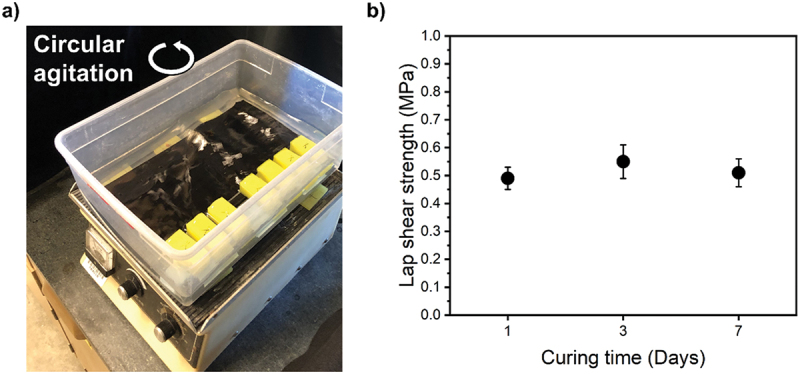
Graphical representation of a) the moving water experiment, and b) adhesion performance of PVCS after constant water agitation.

In addition to water temperature changes, real-world underwater environments often experience dynamic movement due to currents, waves, or tidal forces. Applying adhesives to such surfaces does not always benefit from immobile substrates. We designed a simplified experiment to simulate these conditions and assess the performance of our optimized adhesive. In this experiment, the water container was placed on an orbital shaker set at 50 revolutions per minute, to replicate the effects of dynamic movement (Figure 8a and Movie S1). The adhesive formulation was applied to one substrate in air, then joined to a moving, underwater substrate. No weights were placed on top of the samples during this test. While a decrease in adhesion strength was anticipated due to less precise placement, water movement, and an absence of curing weights, the observed decrease was less than 30% compared to the values obtained in still water after up to 1 week of curing (Figure 7) seen here in Figure 8b. This result suggests promising potential for use of PVCS in dynamic underwater environments.

Salinity and pH are also important factors to consider when bonding substrates underwater. Potential applications of PVCS could extend to brackish waters, which have lower salinity than seawater but higher than fresh water [85]. To investigate the impact of reduced salinity on adhesion performance, water salinity was decreased in adhesion studies from a specific gravity of 1.025, typical for seawater, to 1.012, common for brackish waters [86]. This reduction in salinity resulted in a decrease in adhesion, with lap shear strength dropping to 0.50 ± 0.08 MPa, compared to 0.71 ± 0.08 MPa in specific gravity 1.025 artificial seawater (Supplementary Information, Figure S19a). These findings align with previous reports showing a decrease in adhesion when using deionized water instead of artificial seawater (salinity = 35 g/L) [33].

Similarly, water pH values may influence the performance of catechol-containing adhesives. Catechol oxidation is promoted at higher pH values in the presence of oxygen. Conversely, this moiety becomes less reactive under more acidic conditions [87]. Oxidative cross-linking, a primary adhesive mechanism in catecholic species [88], may therefore be impacted by pH variations, affecting the adhesive performance of poly(vinylcatechol-styrene). Figure S19b in the Supplementary Information shows lap shear strength of the optimized formulation cured in waters at pH values of 5, 7, and 9. The highest adhesion was observed at neutral pH (0.71 ± 0.08 MPa), while adhesion decreased at both lower and higher pH levels. Specifically, a lap shear strength of 0.51 ± 0.01 MPa was seen for acidic conditions (pH = 5) and basic conditions yielded 0.49 ± 0.12 MPa (pH = 9).

### 3.6. 90° peel tests

Joints are often subjected to a variety of mechanical stresses, particularly in underwater environments. To evaluate the performance of PVCS under different stress conditions, 90° peel tests were conducted. Coated steel was used for the rigid substrate. Etched Teflon® and polypropylene provided the complementary flexible adherends. The adhesive was applied underwater between coated steel and Teflon® or coated steel and polypropylene. Joints were cured for 7 days before 90° peel testing.

**Figure 9.**
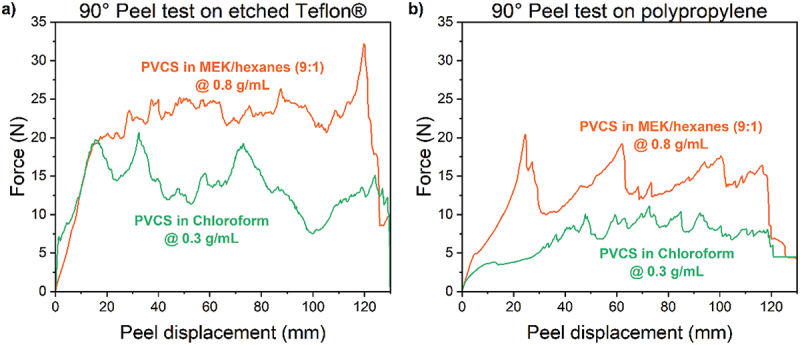
Peel load–displacement curves for PVCS formulations on a) etched Teflon® and b) polypropylene substrates.

Bonding used both the control formulation (PVCS dissolved in chloroform at 0.3 g/mL) and the optimized formulation (PVCS dissolved in MEK/hexanes (9:1) at 0.8 g/mL). Table 3 presents the average peel loads, peel strengths, and maximum loads. The optimized formulation demonstrated superior peel strength compared to the 0.3 g/mL PVCS in chloroform formulation. Peel strengths were greater when etched Teflon® was used for the flexible adherend, as compared to polypropylene, likely due to the scuffed texture of the etched Teflon® substrate, which facilitated adhesive bonding. When using the optimized formulation on etched Teflon®, the peel strength was 0.8 ± 0.2 N/mm, compared to 0.5 ± 0.1 N/mm with the control formulation. In contrast, when polypropylene was used for the flexible adherend with the MEK/hexanes (9:1) formulation, the peel strength was 0.5 ± 0.1 N/mm, compared to 0.3 ± 0.1 N/mm for the chloroform formulation. Figure 9 presents the peel load versus displacement plots for both substrates (etched Teflon®, Figure 9a; and polypropylene, Figure 9b) with the different formulations. The PVCS in MEK/hexanes formulation resulted in an average peel load of 20 ± 4 N on etched Teflon® and 13 ± 1 N on polypropylene. In comparison, the PVCS in chloroform formulation yielded average peel loads of 13 ± 3 N and 8 ± 1 N for Teflon® and polypropylene, respectively. Visual inspection revealed that polypropylene substrates exhibited wrinkling and swelling after 7 days of exposure to the chloroform-based adhesive formulation.

**Table 3.** 90° peel test results for PVCS formulations on different substrates.

Flexible substrate	Adhesive	Avg. peel load (N)	Peel strength (N/mm)	Maximum load (N)
Etched Teflon®	PVCS in MEK/Hexanes (9:1) at 0.8 g/mL	20 ± 4	0.80 ± 0.17	38 ± 4
	PVCS in Chloroform at 0.3 g/mL	13 ± 3	0.51 ± 0.13	25 ± 3
Polypropylene	PVCS in MEK/Hexanes (9:1) at 0.8 g/mL	13 ± 1	0.51 ± 0.05	26 ± 3
	PVCS in Chloroform at 0.3 g/mL	8 ± 1	0.31 ± 0.06	16 ± 4

## 4. Conclusions

This study investigated and tuned factors crucial for the scalability and practical use of catechol-containing adhesives, when working underwater. We evaluated the influence of solvent, polymer concentration, and filler addition on the performance of a poly(vinylcatechol-styrene) copolymer system. Benchmarking experiments demonstrated that this polymer outperformed commercial epoxies, cyanoacrylates, and polyurethanes in several cases. Furthermore, the PVCS adhesive maintained strong adhesion under various water temperatures, extended durations, flow conditions, and varying pH and salinity levels, indicating viable uses in diverse environments. This work is placed within a context where the most recent adhesion chemistry innovations – acrylates, epoxies, and urethanes – were introduced over 50 years ago. Data presented here help to position mussel-mimetic adhesive polymers as versatile candidates for market introduction, particularly for underwater applications.

## Supplementary Material

Supplemental Material

Supplemental Material
