# Supplementary material for: Formulation of catechol-containing adhesives for enhanced underwater bonding and workability
Source: Sci Technol Adv Mater. 2025 Mar 6;26(1):2467617. doi: 10.1080/14686996.2025.2467617 (PMC11892047; doi:10.1080/14686996.2025.2467617)
Supplement: Supplemental Material [file TSTA_A_2467617_SM0732.docx]

**Supplementary Information for:**

**Formulation of Catechol-Containing Adhesives for Enhanced Underwater Bonding and Workability**

Cindy L. Atencio-Martinez^a^, Alexandre Lancelot^a^ and Jonathan J. Wilker^a, b,*^

*^a^ James Tarpo Jr. and Margaret Tarpo Department of Chemistry, Purdue University, 560 Oval Drive, West Lafayette, IN 47907-2084, USA*

*^b^School of Materials Engineering, Purdue University, 701 W. Stadium Avenue, West Lafayette, IN 47907-2045, USA*

[**Figure S1.** ^1^H NMR spectrum of poly(vinylcatechol-styrene). S3](#_Toc188798354)

[**Figure S2.** GPC chromatogram of poly(3,4-dimethoxystyrene-styrene). S4](#_Toc188798355)

[**Figure S3.** Influence of solvent selection on the underwater adhesion of poly(vinylcatechol-styrene) at a concentration of 0.3 g/mL after 3 days. S5](#_Toc188798356)

[**Figure S4.** Effect of hexanes addition to the MEK/hexanes mixture on the underwater adhesion of poly(vinyl catechol-styrene). S6](#_Toc188798357)

[**Figure S5.** ^1^H NMR spectrum of water uptake in PVCS adhesive dissolved in chloroform following lap shear testing. S7](#_Toc188798358)

[**Figure S6.** ^1^H NMR spectrum of water uptake in PVCS adhesive dissolved in MEK following lap shear testing. S8](#_Toc188798359)

[**Figure S7.** ^1^H NMR spectrum of water uptake in PVCS adhesive dissolved in MEK/hexanes (9:1) following lap shear testing. S9](#_Toc188798360)

[**Figure S8**. ^1^H NMR spectrum of water uptake in PVCS adhesive dissolved in chloroform. Adhesive bonds were dried at ambient conditions for 1 hour, and samples were retrieved after lap shear testing. S10](#_Toc188798361)

[**Figure S9**. ^1^H NMR spectrum of water uptake in PVCS adhesive dissolved in MEK. Adhesive bonds were dried at ambient conditions for 1 hour, and samples were retrieved after lap shear testing. S11](#_Toc188798362)

[**Figure S10**. ^1^H NMR spectrum of water uptake in PVCS adhesive dissolved in MEK/hexanes (9:1). Adhesive bonds were dried at ambient conditions for 1 hour, and samples were retrieved after lap shear testing. S12](#_Toc188798363)

[**Figure S11.** Water contact angle measurements on a) coated steel, b) PVCS adhesive layer, and c) coated steel after scraping a PVCS adhesive layer. S13](#_Toc188798364)

[**Figure S12.** Linear plot of viscosity versus shear rate of PCS solutions at varying concentrations. S14](#_Toc188798365)

[**Figure S13.** Viscosity versus concentration of poly(vinylcatechol-styrene) and polystyrene solutions at a shear rate of 10 s^-1^. S15](#_Toc188798366)

[**Figure S14.** Well-dispersed solutions of poly(vinylcatechol-styrene) (PVCS) and fillers in MEK/hexanes (9:1). a) Photographs showing PVCS alone, PVCS + 2 wt.% CaCO_3_, and PVCS + 6 wt.% ABR, demonstrating even dispersion and color changes upon adding fillers –whitening with the addition of calcium carbonate and blackening with the addition of rubber powder. Optical micrographs of b) PVCS, c) PVCS + 2 wt.% CaCO_3_, and d) PVCS + 6 wt.% ABR confirm the uniform dispersion of fillers in the adhesive matrix. S16](#_Toc188798367)

[**Figure S15.** Images of failed substrates. a) Adhesive failure in coated steel bonded to polyurethane substrates, and b) cohesive failure in aluminum substrates. S17](#_Toc188798368)

[**Figure S16.** Representative lap shear curves for coated steel bonded to polyurethane substrates using a) PVCS adhesive, b) PVCS + 2 wt. % CaCO3, and c) PVCS + 6 wt. % ABR. S18](#_Toc188798369)

[**Figure S17.** Representative lap shear curves for polished aluminum substrates bonded to polished aluminum using a) PVCS adhesive, b) PVCS + 2 wt. % CaCO3, and c) PVCS + 6 wt. % ABR. S19](#_Toc188798370)

[**Figure S18**. SEM micrograph of isolated CaCO_3_ particles. S20](#_Toc188798371)

[**Figure S19.** Study of the effect of a) water salinity and b) pH on the underwater adhesion strength of PVCS. S21](#_Toc188798372)


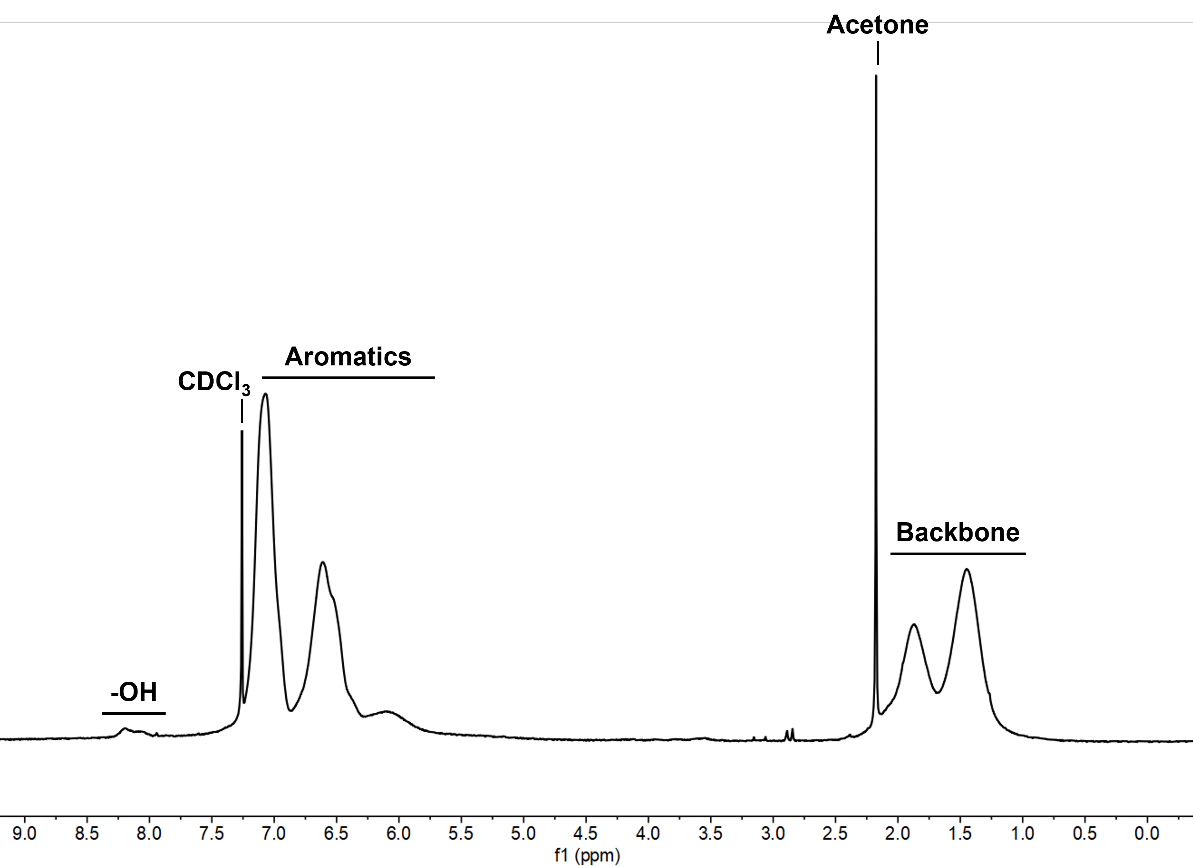


**Figure S1.** ^1^H NMR spectrum of poly(vinylcatechol-styrene).


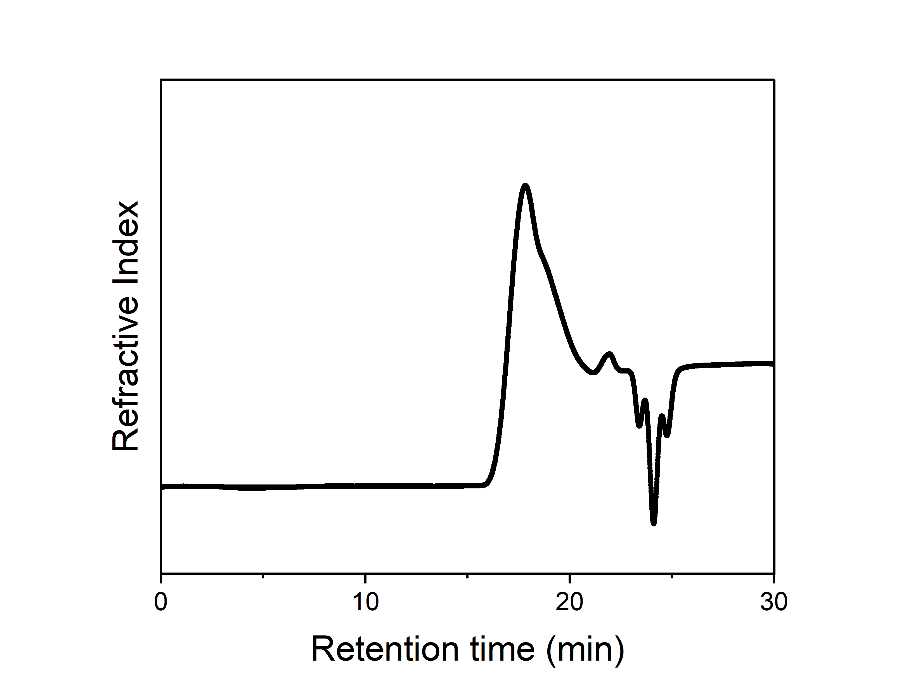


**Figure S2.** GPC chromatogram of poly(3,4-dimethoxystyrene-styrene).


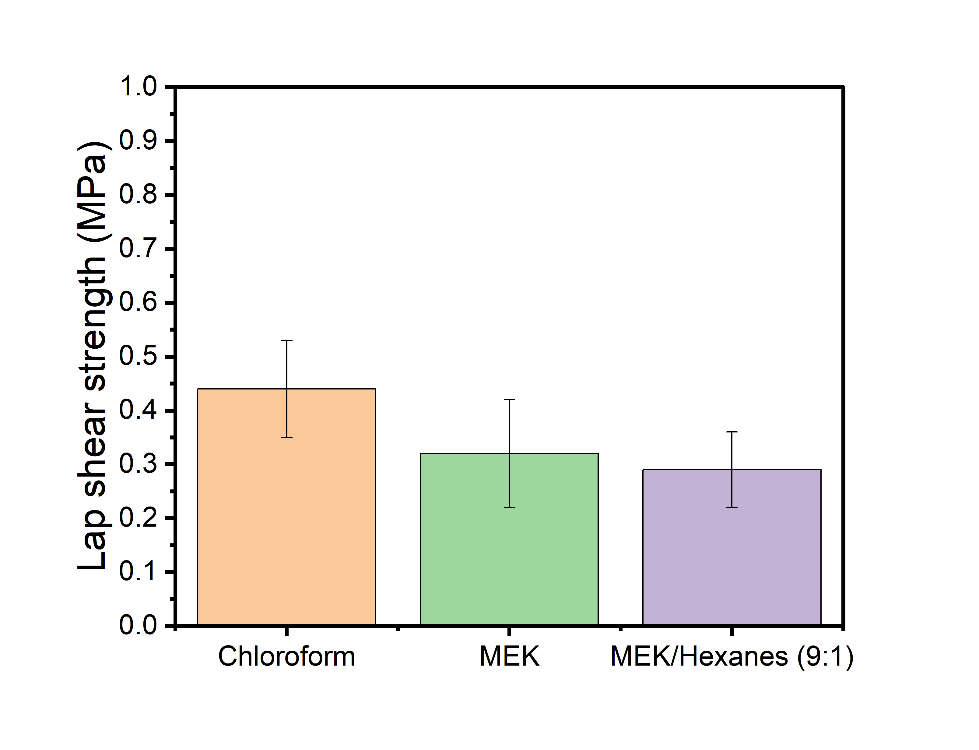


**Figure S3.** Influence of solvent selection on the underwater adhesion of poly(vinylcatechol-styrene) at a concentration of 0.3 g/mL after 3 days.


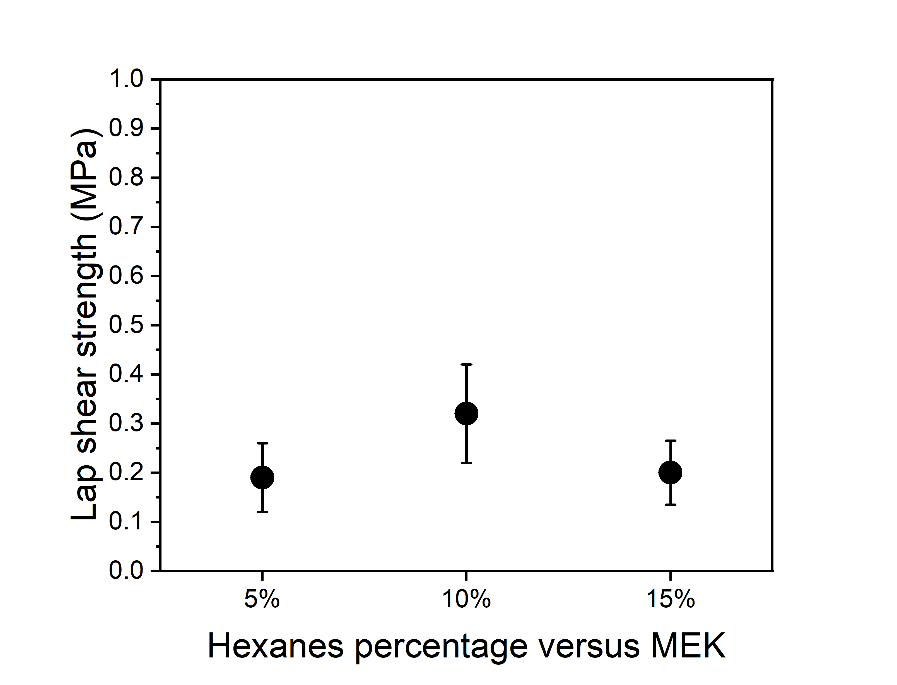


**Figure S4.** Effect of hexanes addition to the MEK/hexanes mixture on the underwater adhesion of poly(vinyl catechol-styrene).


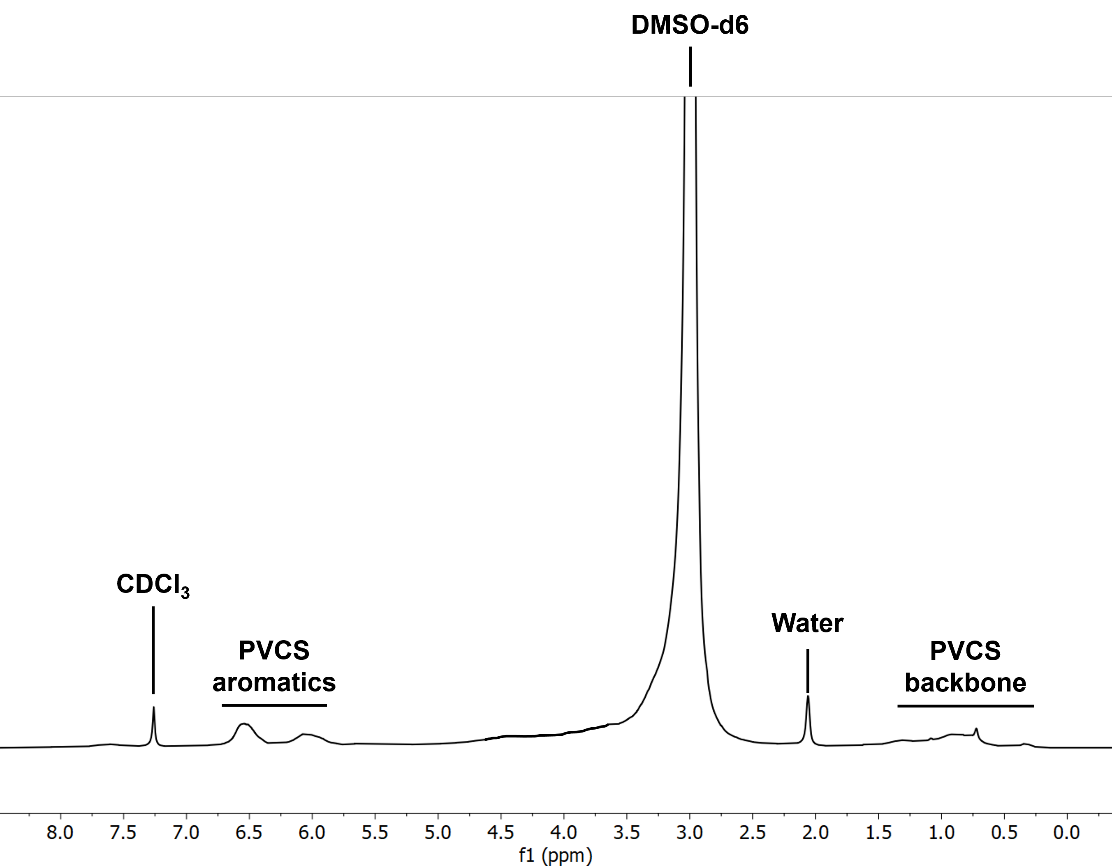


**Figure S5.** ^1^H NMR spectrum of water uptake in PVCS adhesive dissolved in chloroform following lap shear testing.


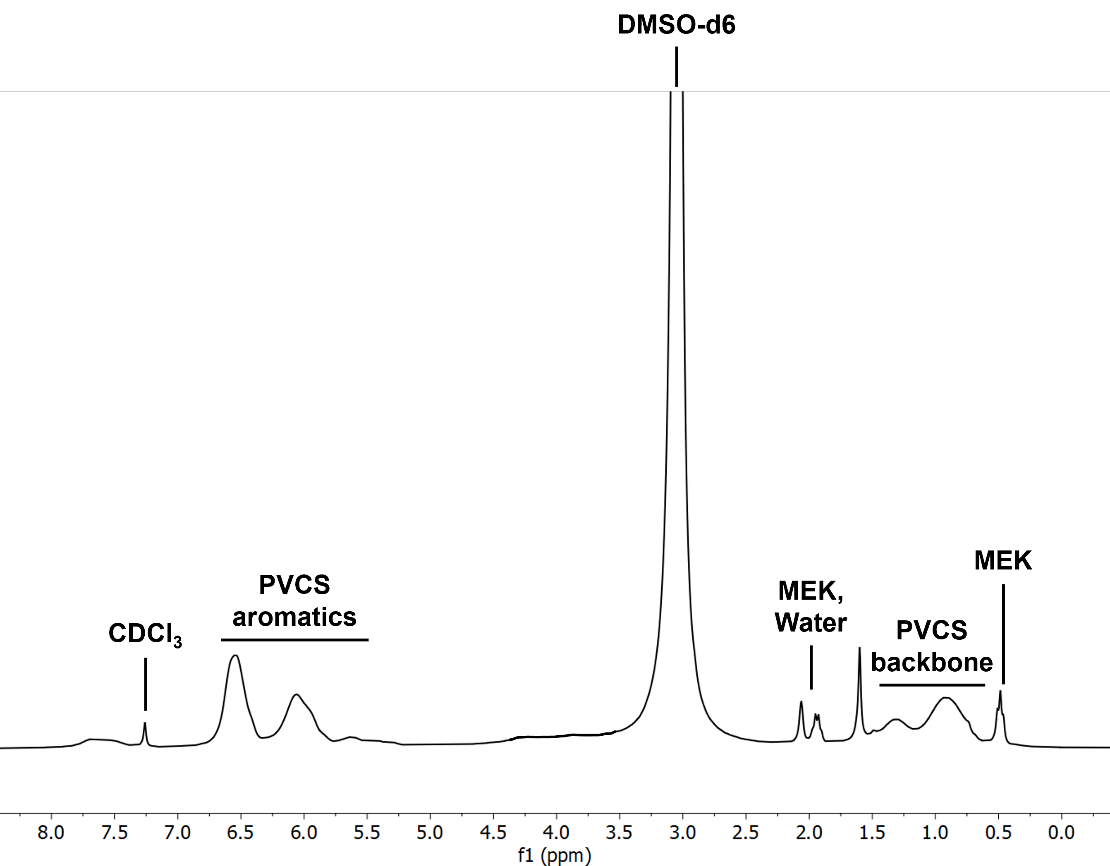


**Figure S6.** ^1^H NMR spectrum of water uptake in PVCS adhesive dissolved in MEK following lap shear testing.


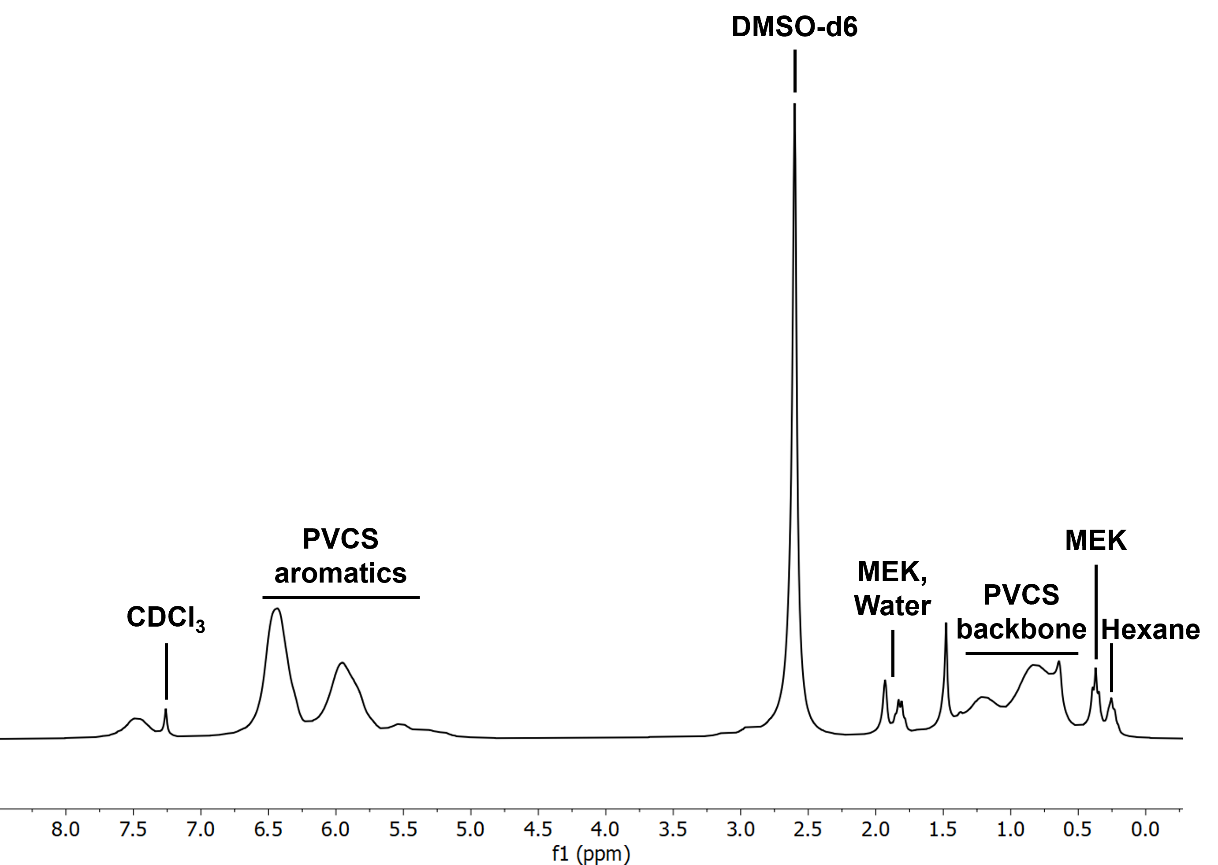


**Figure S7.** ^1^H NMR spectrum of water uptake in PVCS adhesive dissolved in MEK/hexanes (9:1) following lap shear testing.


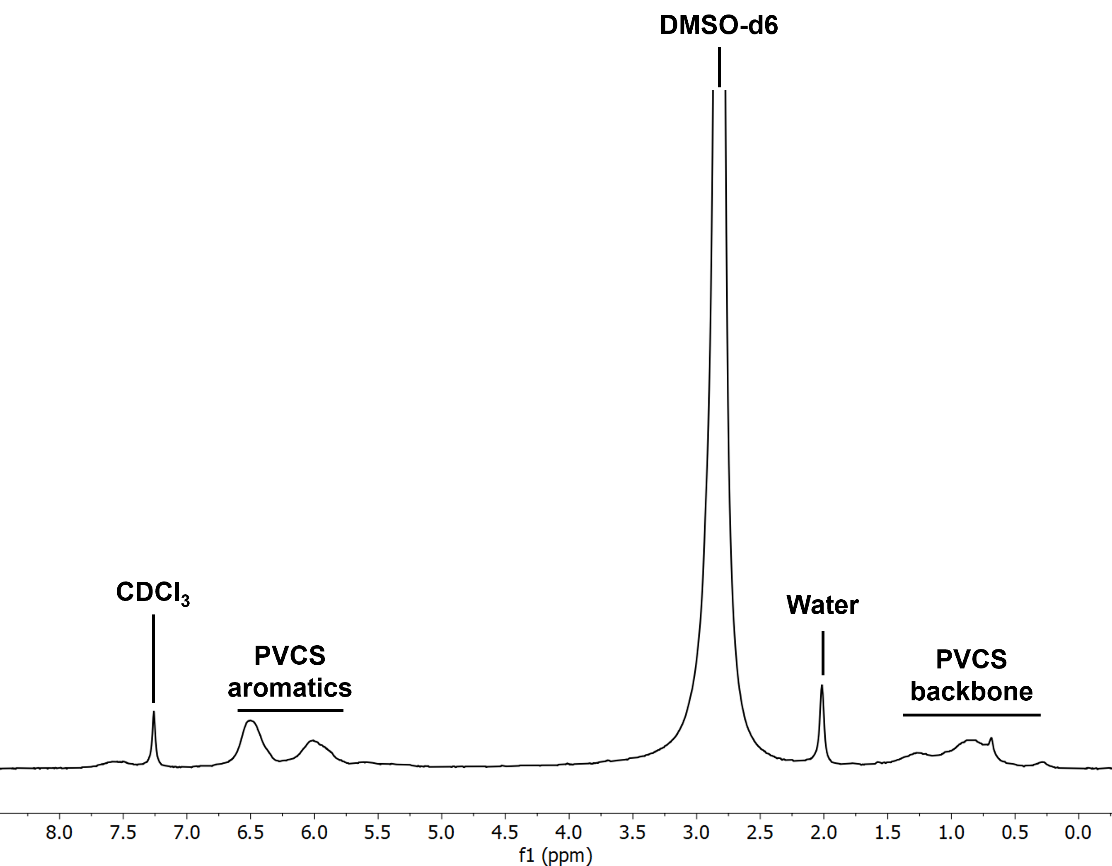


**Figure S8**. ^1^H NMR spectrum of water uptake in PVCS adhesive dissolved in chloroform. Adhesive bonds were dried at ambient conditions for 1 hour, and samples were retrieved after lap shear testing.


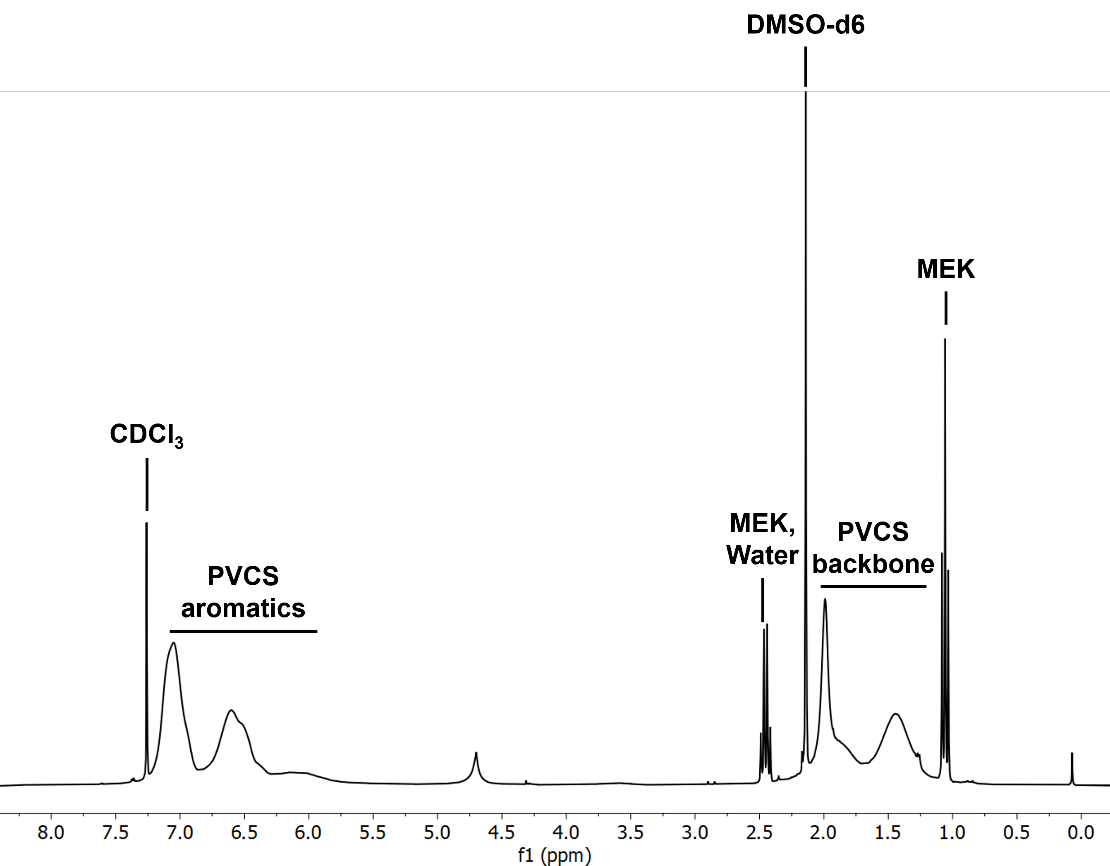


**Figure S9**. ^1^H NMR spectrum of water uptake in PVCS adhesive dissolved in MEK. Adhesive bonds were dried at ambient conditions for 1 hour, and samples were retrieved after lap shear testing.


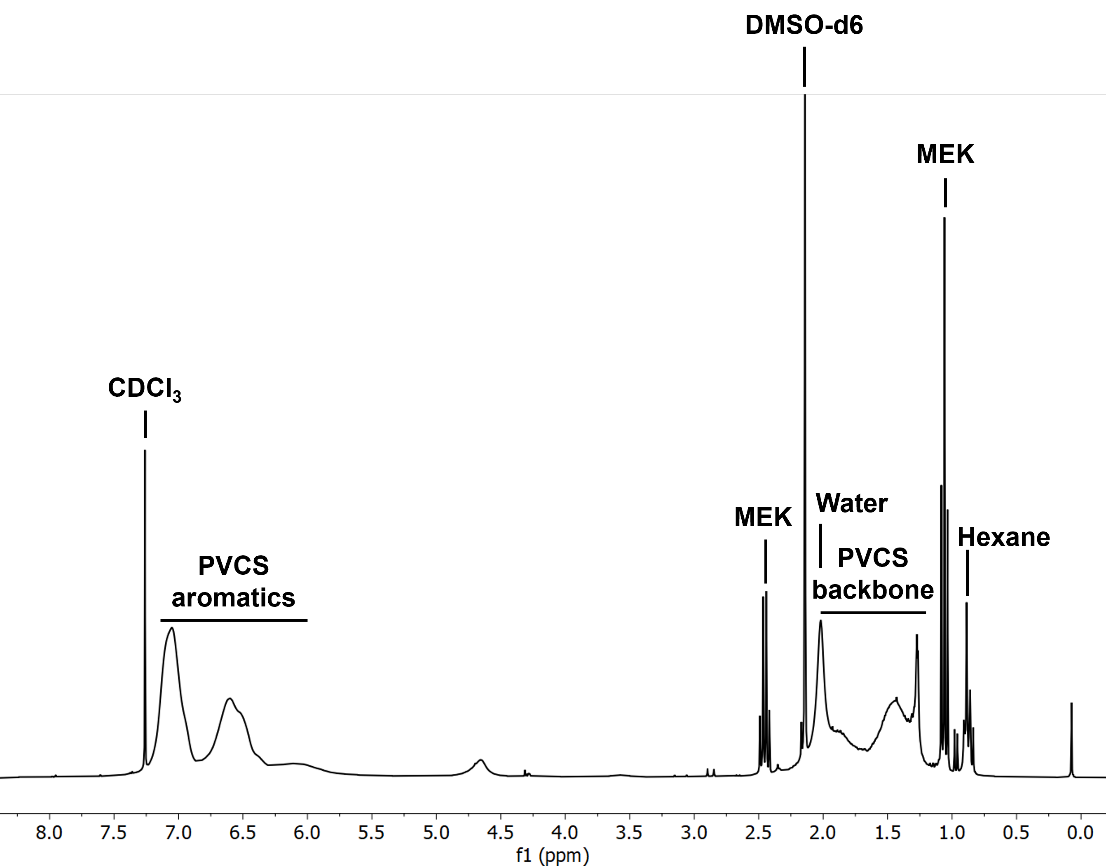


**Figure S10**. ^1^H NMR spectrum of water uptake in PVCS adhesive dissolved in MEK/hexanes (9:1). Adhesive bonds were dried at ambient conditions for 1 hour, and samples were retrieved after lap shear testing.


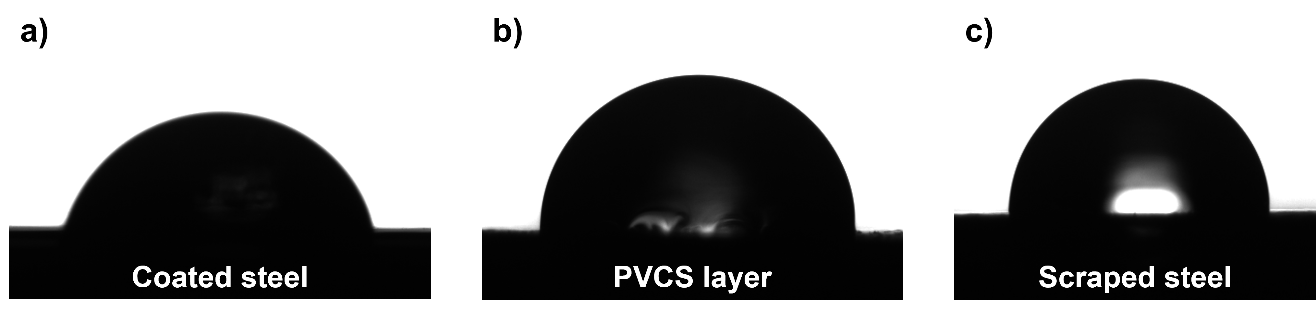


**Figure S11.** Water contact angle measurements on a) coated steel, b) PVCS adhesive layer, and c) coated steel after scraping a PVCS adhesive layer.


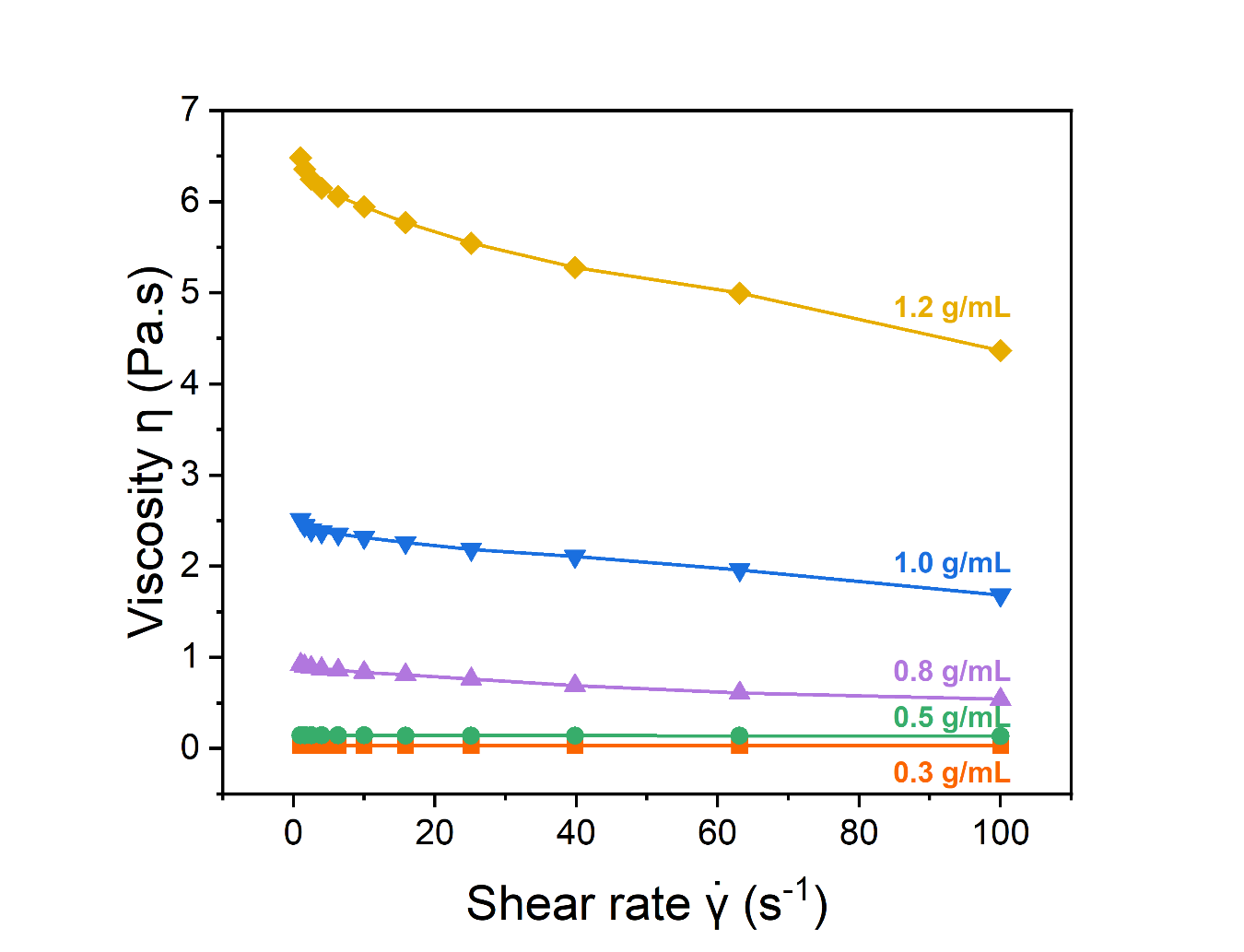


**Figure S12.** Linear plot of viscosity versus shear rate of PCS solutions at varying concentrations.


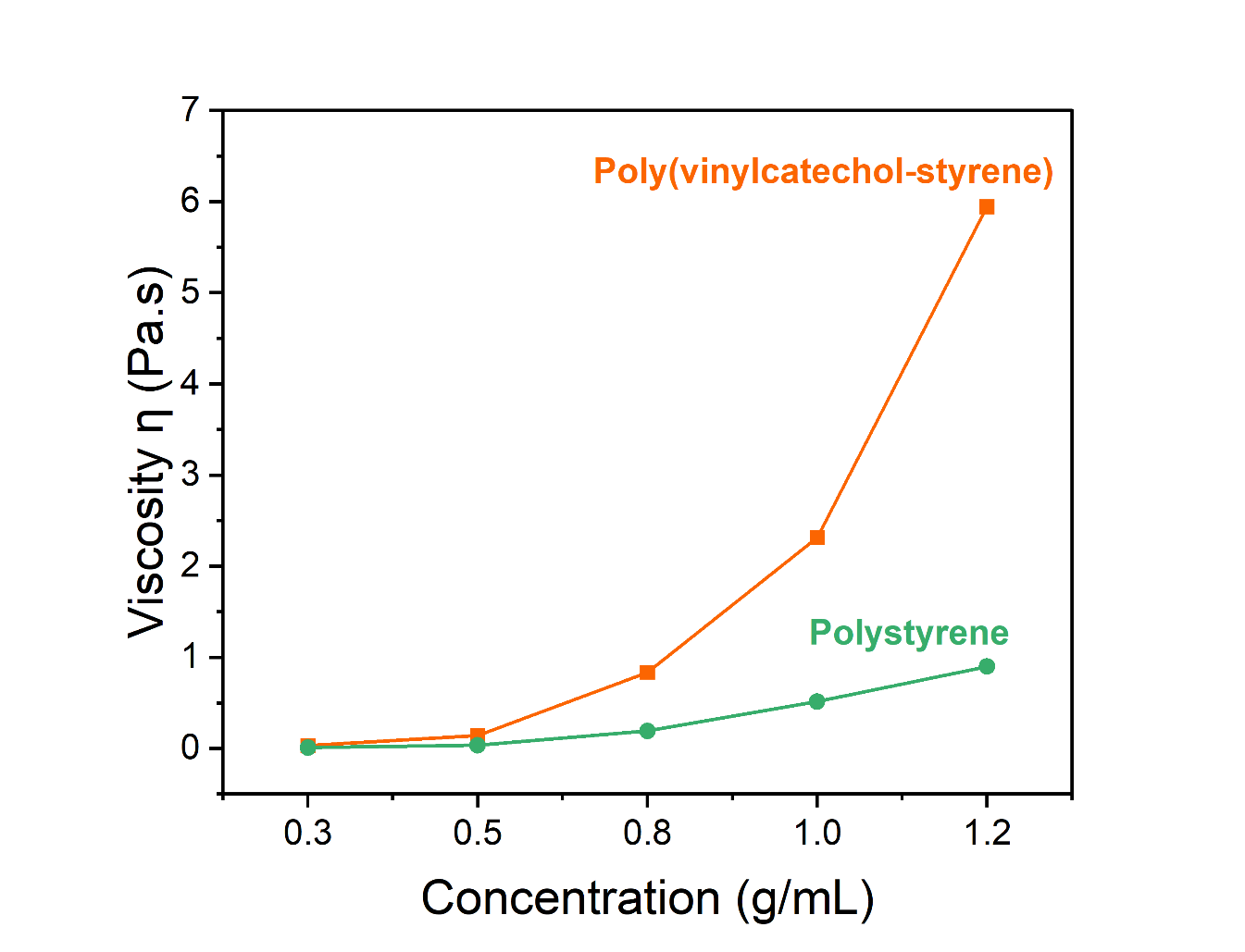


**Figure S13.** Viscosity versus concentration of poly(vinylcatechol-styrene) and polystyrene solutions at a shear rate of 10 s^-1^.


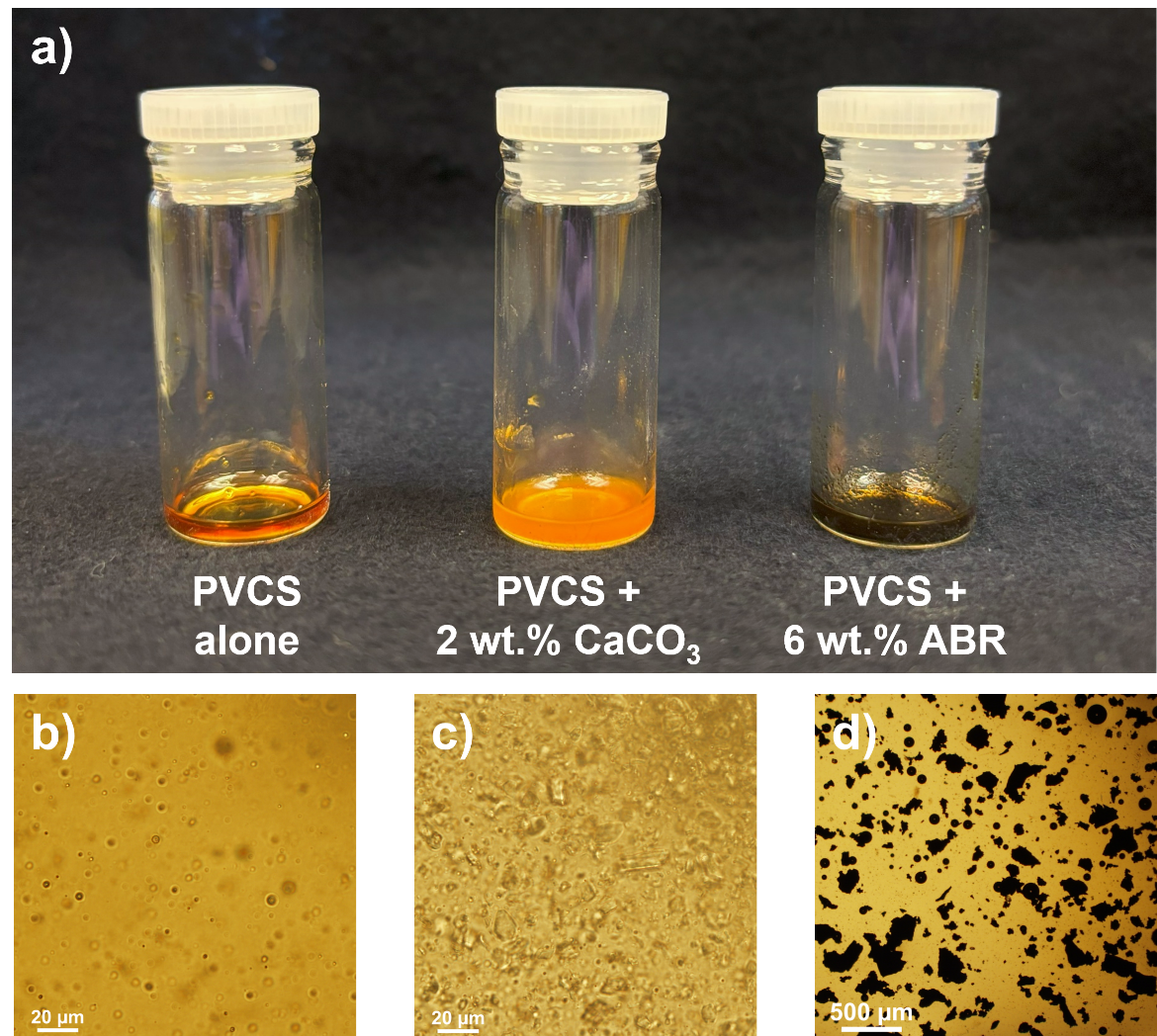


**Figure S14.** Well-dispersed solutions of poly(vinylcatechol-styrene) (PVCS) and fillers in MEK/hexanes (9:1). a) Photographs showing PVCS alone, PVCS + 2 wt.% CaCO_3_, and PVCS + 6 wt.% ABR, demonstrating even dispersion and color changes upon adding fillers –whitening with the addition of calcium carbonate and blackening with the addition of rubber powder. Optical micrographs of b) PVCS, c) PVCS + 2 wt.% CaCO_3_, and d) PVCS + 6 wt.% ABR confirm the uniform dispersion of fillers in the adhesive matrix.


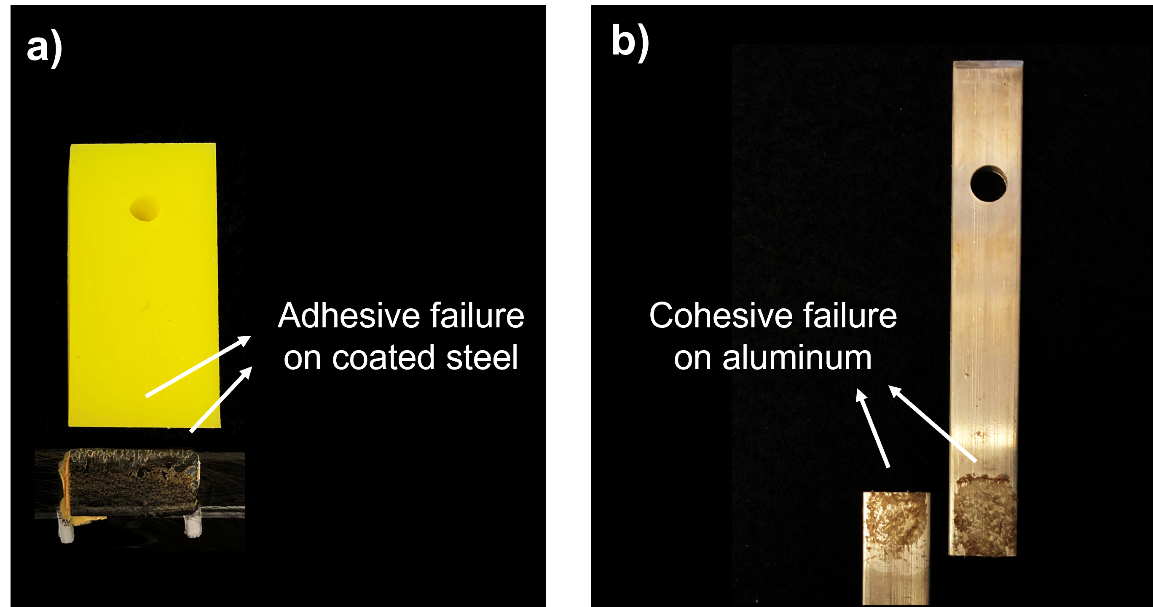


**Figure S15.** Images of failed substrates. a) Adhesive failure in coated steel bonded to polyurethane substrates, and b) cohesive failure in aluminum substrates.


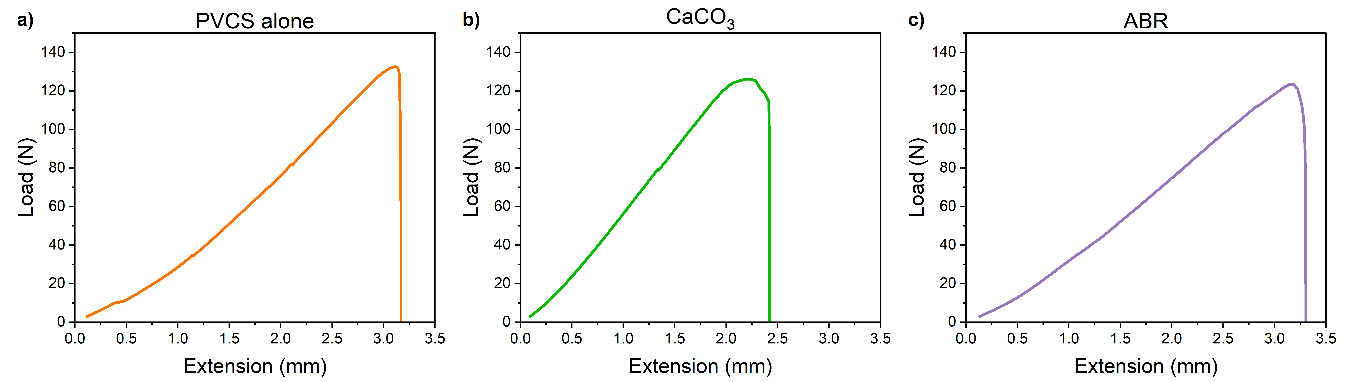


**Figure S16.** Representative lap shear curves for coated steel bonded to polyurethane substrates using a) PVCS adhesive, b) PVCS + 2 wt. % CaCO3, and c) PVCS + 6 wt. % ABR.


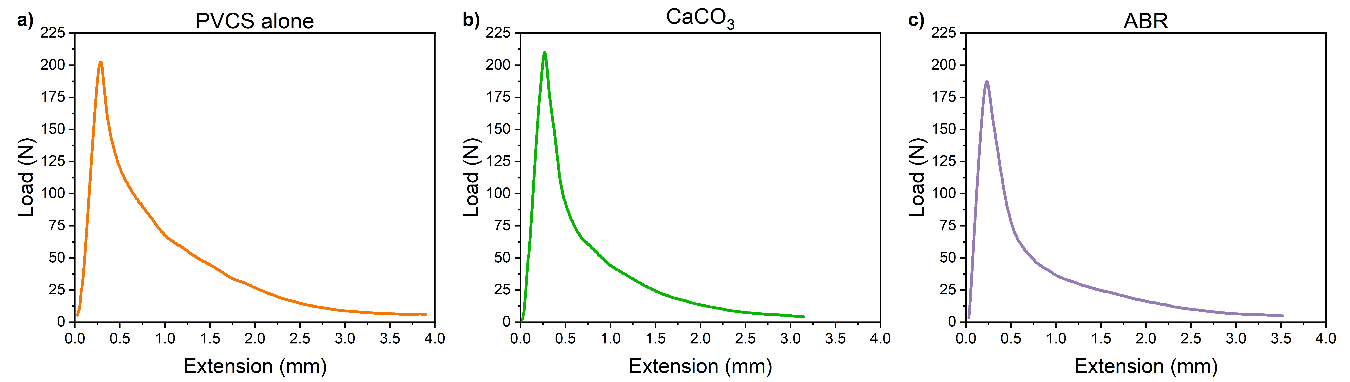


**Figure S17.** Representative lap shear curves for polished aluminum substrates bonded to polished aluminum using a) PVCS adhesive, b) PVCS + 2 wt. % CaCO3, and c) PVCS + 6 wt. % ABR.


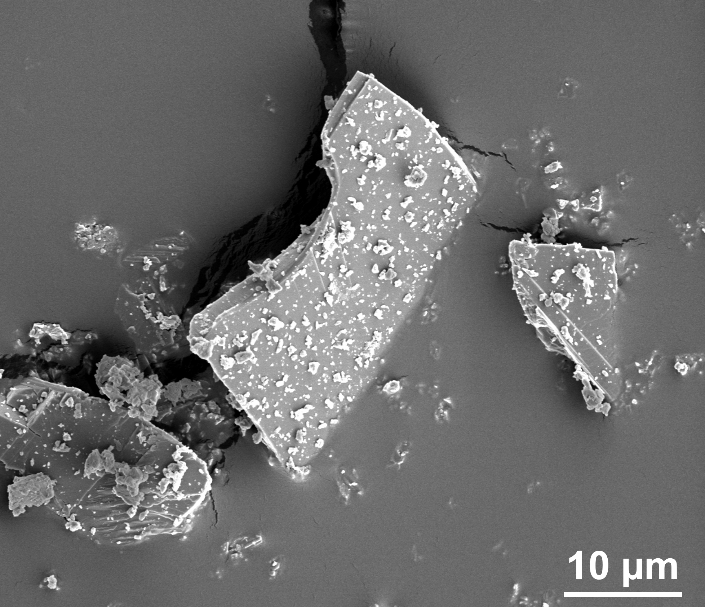


**Figure S18**. SEM micrograph of isolated CaCO_3_ particles.


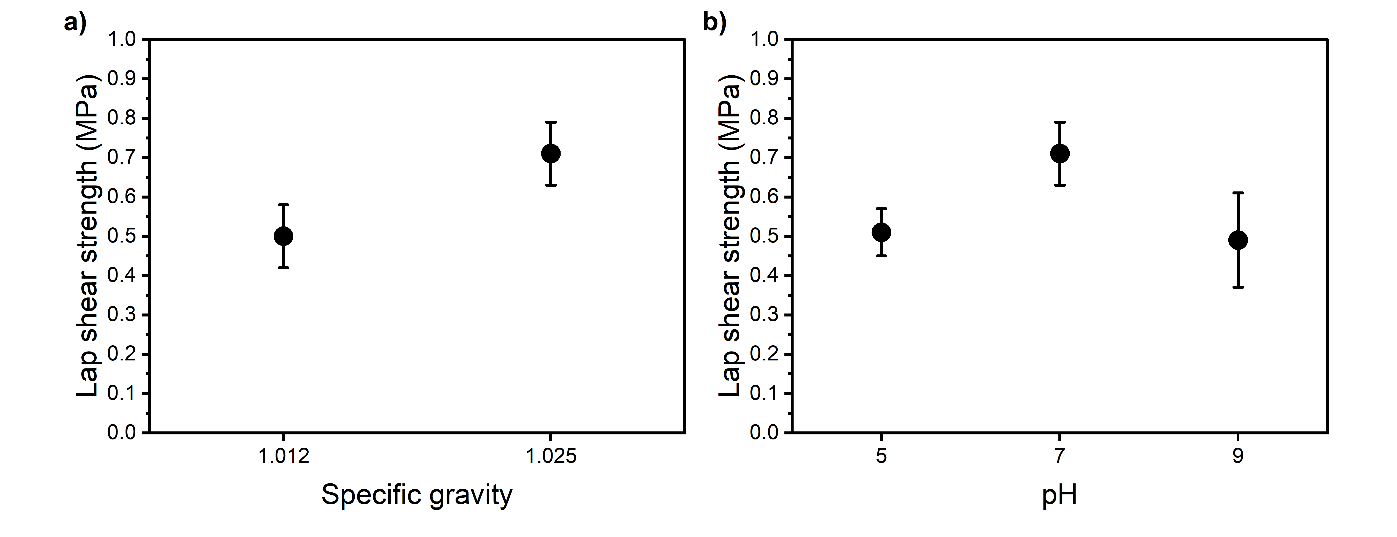


**Figure S19.** Study of the effect of a) water salinity and b) pH on the underwater adhesion strength of PVCS.
